# Altered Atlas of Exercise-Responsive MicroRNAs Revealing miR-29a-3p Attacks Armored and Cold Tumors and Boosts Anti-B7-H3 Therapy

**DOI:** 10.34133/research.0590

**Published:** 2025-01-22

**Authors:** Jie Mei, Zhiwen Luo, Yun Cai, Renwen Wan, Zhiwen Qian, Jiahui Chu, Yaying Sun, Yuxin Shi, Ying Jiang, Yan Zhang, Yongmei Yin, Shiyi Chen

**Affiliations:** ^1^ Department of Oncology, The First Affiliated Hospital of Nanjing Medical University, Nanjing 210029, China.; ^2^ The First Clinical Medicine College, Nanjing Medical University, Nanjing 211166, China.; ^3^ Department of Sports Medicine, Huashan Hospital Affiliated to Fudan University, Shanghai 200040, China.; ^4^ Department of Central Laboratory, Changzhou Jintan First People’s Hospital, Jiangsu University, Changzhou 213200, China.; ^5^ Departments of Gynecology, Wuxi Maternal and Child Health Care Hospital, Wuxi Medical Center, Nanjing Medical University, Wuxi 214023, China.; ^6^ Department of Sports Medicine, Shanghai General Hospital, Shanghai Jiao Tong University School of Medicine, Shanghai 200080, China.; ^7^ Department of Oncology, The Affiliated Wuxi People’s Hospital of Nanjing Medical University, Wuxi People’s Hospital, Wuxi Medical Center, Nanjing Medical University, Nanjing 211166, China.; ^8^ Department of Gynecology, The Obstetrics and Gynecology Hospital Affiliated to Jiangnan University, Wuxi 214023, China.; ^9^ Jiangsu Key Lab of Cancer Biomarkers, Prevention and Treatment, Collaborative Innovation Center for Personalized Cancer Medicine, Nanjing Medical University, Nanjing 211166, China.; ^10^ Collaborative Innovation Center for Cancer Personalized Medicine, Nanjing Medical University, Nanjing, 211166, China.

## Abstract

Increasing evidence has shown that physical exercise remarkably inhibits oncogenesis and progression of numerous cancers and exercise-responsive microRNAs (miRNAs) exert a marked role in exercise-mediated tumor suppression. In this research, expression and prognostic values of exercise-responsive miRNAs were examined in breast cancer (BRCA) and further pan-cancer types. In addition, multiple independent public and in-house cohorts, in vitro assays involving multiple, macrophages, fibroblasts, and tumor cells, and in vivo models were utilized to uncover the tumor-suppressive roles of miR-29a-3p in cancers. Here, we reported that miR-29a-3p was the exercise-responsive miRNA, which was lowly expressed in tumor tissues and associated with unfavorable prognosis in BRCA. Mechanistically, miR-29a-3p targeted macrophages, fibroblasts, and tumor cells to down-regulate B7 homolog 3 (B7-H3) expression. Single-cell RNA sequencing (scRNA-seq) and cytometry by time-of-flight (CyTOF) demonstrated that miR-29a-3p attacked the armored and cold tumors, thereby shaping an immuno-hot tumor microenvironment (TME). Translationally, liposomes were developed and loaded with miR-29a-3p (lipo@miR-29a-3p), and lipo@miR-29a-3p exhibited promising antitumor effects in a mouse model with great biocompatibility. In conclusion, we uncovered that miR-29a-3p is a critical exercise-responsive miRNA, which attacked armored and cold tumors by inhibiting B7-H3 expression. Thus, miR-29a-3p restoration could be an alternative strategy for antitumor therapy.

## Introduction

Although the risk of cancer-related death has declined continuously since the 1990s, cancer is still a considerable public health concern worldwide [1]. It has been well recognized that mutations in functional genes support the oncogenesis and cancer progression as oncogenic drivers [2]. However, for a given individual, the cancer risk is consumedly enhanced with exposure to “genotoxic” lifestyles and habits such as tobacco consumption, excess alcohol intake, and lack of physical exercise [3,4]. If such “genotoxic” lifestyles and habits promote the carcinogenesis of malignant tumors, there is a reason to assume that the opposite may also be true: Exposure to “physiological” lifestyles and habits might prevent oncogenesis and progression. More and more research demonstrates that individuals who consistently engage in regular exercise seem to significantly reduce their chances of developing noncommunicable diseases, including cancerous diseases [5–8].

Over the past 3 decades, multiple observational studies have revealed that physical exercise remarkably decreases the risk of the oncogenesis of numerous cancers [9–11]. In addition, preliminary studies indicate that proper exercise after the diagnosis of given solid tumors could delay the cancer progression, decrease tumor-related deaths, and enhance the life quality of cancer patients [12,13]. Despite that observational data have linked physical activity to decreases in the oncogenesis and progression of certain cancers, the molecular mechanisms underlying the potential antitumor effects of physical activity have not been well understood. Numerous studies have attempted to explain the mechanisms of physical exercise against cancer, and several targets, such as interleukin-15 (IL-15) and squalene epoxidase (SQLE), have been revealed [7,14], but it has been uncovered that physical activity participates in the reprogramming of the tumor microenvironment (TME) [15,16].

MicroRNA (miRNA) is a class of small noncoding RNA molecules that play crucial roles in controlling gene expression [17]. Exercise-responsive miRNAs refer to miRNAs whose expression levels are regulated by physical exercise [18]. Increasing evidence has shown that exercise-responsive miRNAs exert critical roles in human diseases. The most well-known function of exercise-responsive miRNAs is their impact on cardiovascular health by regulating gene expression in the cardiovascular system [19,20]. For example, the expression of miR-210 and miR-133a is regulated by exercise and related to various heart diseases [21,22]. The close relationship between exercise-responsive miRNAs and malignant tumors is also conspicuous. Exercise can modulate the cancerigenesis and progression of tumors by regulating the expression levels of tumor-related miRNAs [23]. Thus, deepening our understanding of the specific mechanisms by which exercise-responsive miRNAs act in the development of tumors can provide novel insights for cancer management and treatment.

In the current research, breast cancer (BRCA) with the highest morbidity was used as main research carrier and pan-cancer enlargement was also conducted. We proposed a novel insight based on exercise-responsive miRNAs to explain the effects of exercise on the TME. Exercise-responsive circulating miR-29a-3p attacked armored and cold tumors by inhibiting B7 homolog 3 (B7-H3) expression and then promoted the infiltration of tumor-infiltrating immune cells (TIICs), thereby boosting anti-B7-H3 therapeutic response. Due to ease of preparation, strong translational potential, excellent biocompatibility, and high encapsulation efficiency of liposomes, we developed a novel biomaterial lipo@miR-29a-3p to simulate the benefits of exercise. Overall, this study revealed the significant antitumor role of miR-29a-3p, contributing to understanding the molecular mechanisms of exercise-mediated anticancer effects and providing miR-29a-3p as a novel target for refractory armored and cold tumors.

## Results

### Identification of miR-29a-3p as a critical exercise-responsive tumor suppressor

**Fig. 1.**
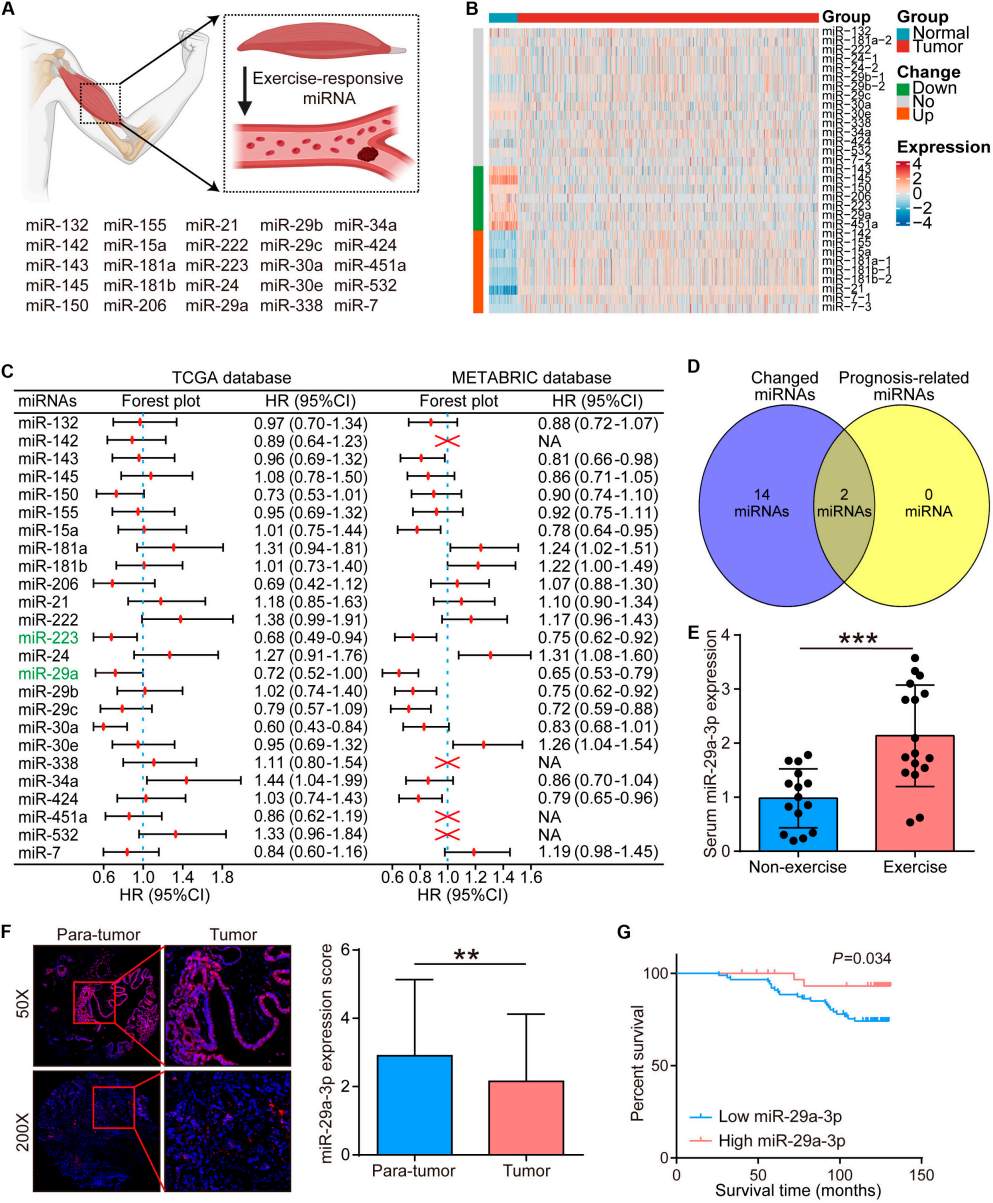
Identification of miR-29a-3p as a clinically relevant exercise-responsive miRNA. (A) Landscape of exercise-responsive miRNAs. (B) Differential expression of exercise-responsive miRNAs between tumor and para-tumor tissues in BRCA. Significance was calculated with Student’s *t* test. (C) Prognostic value in predicting overall survival of exercise-responsive miRNAs in BRCA. Data were obtained from the Kaplan–Meier Plotter tool. Significance was calculated with the log-rank test. (D) Intersection of exercise-responsive miRNAs with differential expression and prognostic value. (E) Validation of miR-29a-3p expression in BRCA patients with and without exercise habit. Data are presented as mean ± SD. Significance was calculated with Mann–Whitney test. ***P < 0.001. (F) Representative images showing miR-29a-3p expression in tumor and para-tumor tissues, along with semiquantitative analysis. Total original magnification, 50× (left) and 200× (right). Data are presented as mean ± SD. Significance was calculated with Mann–Whitney test for (G). *P < 0.05. (G) Prognostic value in predicting overall survival of miR-29a-3p in the in-house BRCA cohort. Significance was calculated with the log-rank test.

A total of 25 miRNAs were identified as exercise-responsive, and more than 70% of studies suggested that concordance in up-regulation in blood obtained from a previous report [24] was included in our study (Fig. 1A). Then, we analyzed the differential expression of these miRNAs between tumor and para-tumor tissues using the TCGA (The Cancer Genome Atlas)–BRCA dataset. The results showed that several miRNAs, including miR-143, miR-145, miR-150, miR-206, miR-223, miR-29a, and miR-451a, were lowly expressed in tumor tissues, while miR-142, miR-155, miR-15a, miR-181a, miR-181b, miR-21, and miR-7, were up-regulated in tumor tissues (Fig. 1B). In addition, the prognostic values of these miRNAs were also explored using the Kaplan–Meier Plotter tool, and only 2 miRNAs (miR-29a and miR-223) exhibited notable prognostic value (Fig. 1C and D). We further conducted a pan-cancer analysis of 2 miRNAs with differential expression and found that miR-29a was conserved in solid tumors and miR-223 was highly expressed in leukemia (Fig. S1A and B). Thus, we chose miR-29a for in-depth research.

Preceding studies have confirmed that miR-29a-3p is the mature of miR-29a and up-regulated during exercise [25–28]. We collected a total of 32 serum and tumor samples from BRCA patients before they received any antitumor therapy (17 with exercise habit and 15 without exercise), and the results showed that miR-29a-3p was highly expressed in both serum exosome and tumor tissues (Fig. 1E and Fig. S2A and B). Next, another cohort consisting of large-scale BRCA tumor samples was used to determine the expression and prognostic value of miR-29a-3p. The results showed that miR-29a-3p was lowly expressed in tumor tissues and high miR-29a-3p was associated with favorable prognosis (Fig. 1F and G). In addition, we detected baseline miR-29a-3p expression in various organs in mouse bearing tumor without exercise, and we found that miR-29a-3p was highly expressed in heart and muscle (Fig. S3A). However, tumor tissues expressed high miR-29a-3p from mouse with exercise (Fig. S3B). Thus, we guessed that exercise promoted circulated miR-29a-3p into tumor tissues. Overall, we identified miR-29a-3p as a significant exercise-responsive miRNA with a potential tumor-suppressive role.

### Immune checkpoint B7-H3 was a target gene of miR-29a-3p

**Fig. 2.**
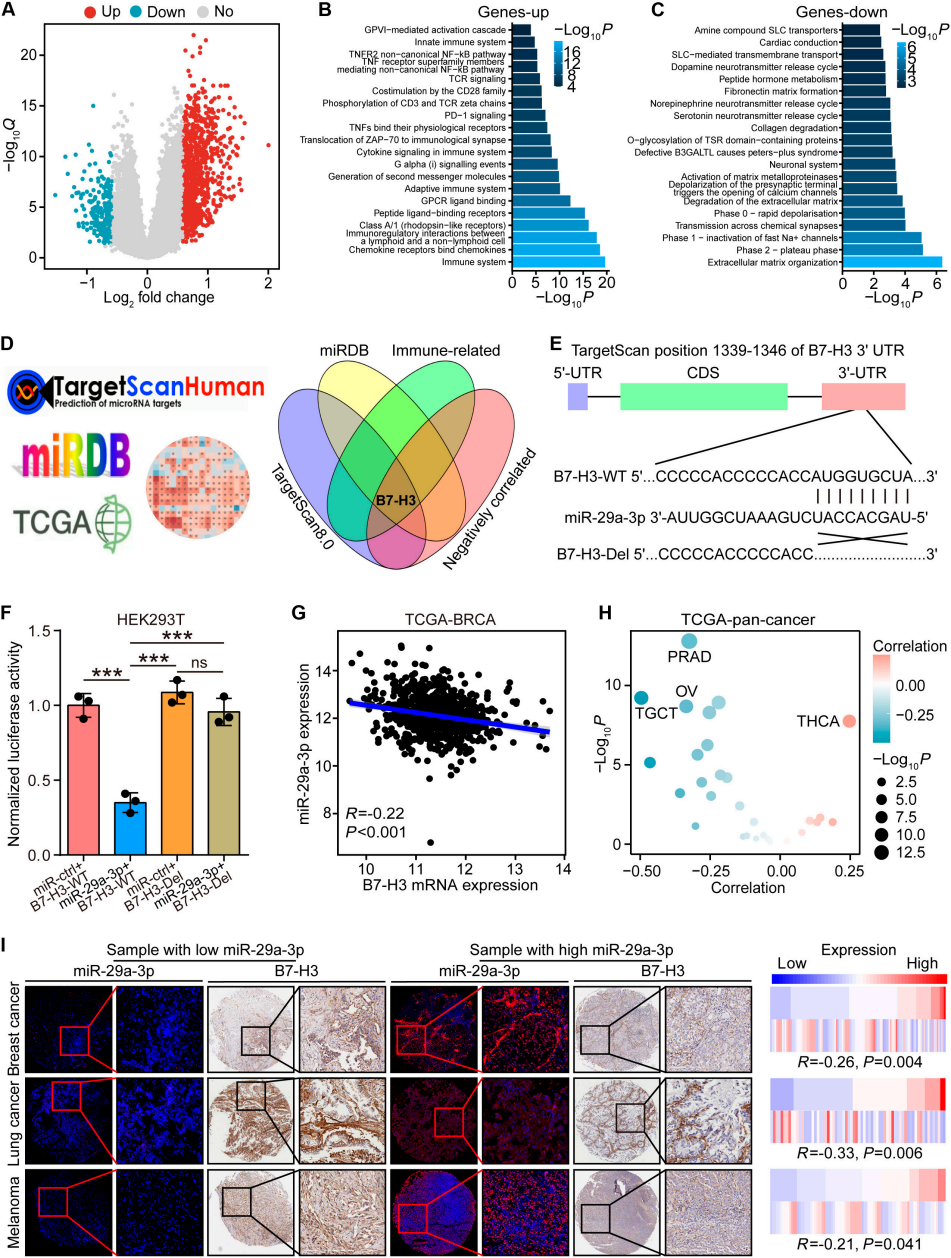
B7-H3 was identified as the target gene of miR-29a-3p. (A) Volcano plot revealing DEGs between low- and high-miR-29a-3p expression with the criterion of FC ≥ 1.5 and adjusted *P* value ≤ 0.05. (B and C) Enrichment of up-regulated and down-regulated genes in the high-miR-29a-3p group using gene sets derived from the Reactome pathway database. (D) Intersection of predicted target genes, immune-related genes, and negatively correlated genes with miR-29a-3p. (E) Diagram revealing the miR-208a-5p binding site in B7-H3 3′-UTR. (F) Luciferase activity in HEK293T cells transfected with WT B7-H3 3′-UTR or mutant B7-H3 3′-UTR and miR-29a-3p or miR-ctrl. Data are presented as mean ± SD. Significance was calculated with one-way ANOVA with Tukey’s multiple-comparison test. ns, no significance. ***P < 0.001. (G and H) Correlation between miR-29a-3p and B7-H3 expression in BRCA and pan-cancer. Data were obtained from the TCGA database. Significance was calculated with Pearson test. (I) Representative images showing B7-H3 expression in tumor tissues with low- and high-miR-29a-3p expression in BRCA, lung cancer, and melanoma, along with semiquantitative analysis. Staining data of miR-29a-3p in lung cancer and melanoma from our previous study [42] were used as controls. Total original magnification, 50× (left) and 200× (right). Significance was calculated with Spearman test.

To understand the biological role of miR-29a-3p in BRCA, we screened the differentially expressed genes (DEGs) between low and high miR-29a-3p expression (Fig. 2A). We found that genes up-regulated in the high miR-29a-3p group were mostly enriched in the “Immune system” term, while genes up-regulated in the low miR-29a-3p group were mostly enriched in the “Extracellular matrix organization” term (Fig. 2B and C). In addition, the target genes of miR-29a-3p were predicted using TargetScan 7.1 and miRDB tools, and these genes were mostly enriched in collagen-related terms (Fig. S4). To identify the target genes of miR-29a-3p, genes were up-regulated in the low miR-29a-3p group in the TCGA-BRCA dataset and the list of immune-related genes [29] was used as qualifications as well, and only B7-H3 was extracted (Fig. 2D).

We next tried to verify the binding of miR-29a-3p and B7-H3 3′-UTR (untranslated region) (Fig. 2E) and performed dual-luciferase activity assays. A notable decline of luciferase activity was shown in B7-H3 3′-UTR wild-type (WT) and miR-29a-3p mimic overexpressed group, and disappeared in miRNA-control and B7-H3 3′-UTR Del groups in HEK293T cells (Fig. 2F). In tumor samples, miR-29a-3p expression was inversely correlated with B7-H3 expression in not only BRCA but also most solid cancer types in the TCGA dataset (Fig. 2G and H). We also validated the correlation between miR-29a-3p and B7-H3 levels, and found that miR-29a-3p was negatively correlated with B7-H3 protein expression in BRCA, lung cancer, and melanoma (Fig. 2I). These results indicated that miR-29a-3p directly targeted B7-H3 in human cancers.

### miR-29a-3p inhibited tumor progression by regulating both stromal cells and tumor cells

**Fig. 3.**
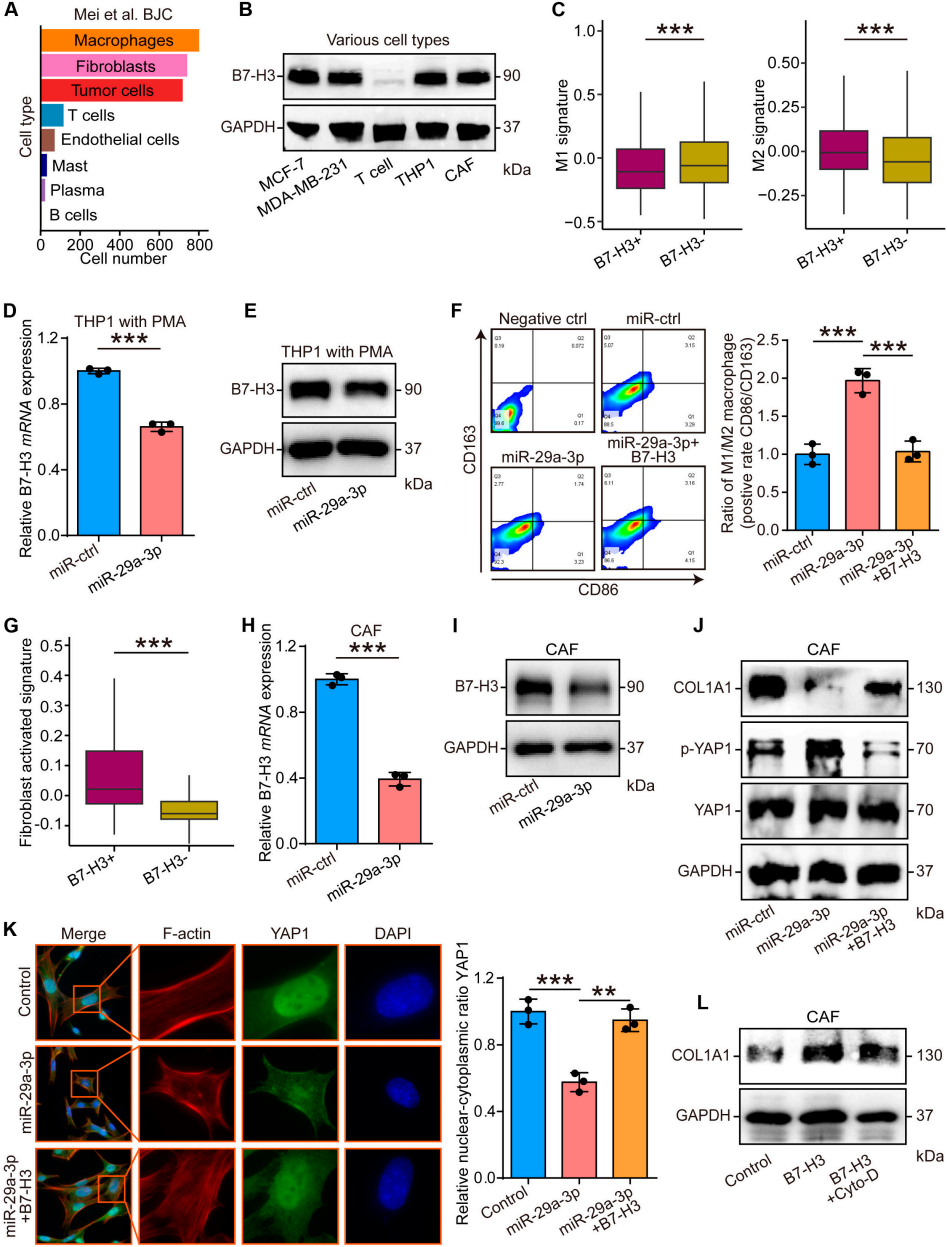
miR-29a-3p inhibited macrophage M2 polarization and collagen expression in CAFs. (A) Number of cell with B7-H3-postive in our published scRNA-seq dataset. (B) Expression of B7-H3 in MCF7, MDAMB231, THP1, CAF, and Jurkat T cells. (C) Level of M1 and M2 signatures in macrophages with B7-H3-positive and B7-H3-negative expression. Significance was calculated with Student’s *t* test. ***P < 0.001. (D and E) Down-regulated B7-H3 in both mRNA and protein levels after miR-29a-3p overexpression in THP1 cells. GAPDH was used as the loading control. Significance was calculated with Student’s *t* test for (D). ***P < 0.001. (F) The expression of M1 marker CD86 and M2 marker CD163 of control, miR-29a-3p-overexpressed, and B7-H3-rescured THP1 cells was examined at 24 h after transfection by flow cytometry. Data are presented as mean ± SD. Significance was calculated with Student’s *t* test. ***P < 0.001. (G) Level of activated signature in fibroblast with B7-H3-positive and B7-H3-negative expression. Significance was calculated with Student’s *t* test. ***P < 0.001. (H and I) Down-regulated B7-H3 in both mRNA and protein levels after miR-29a-3p overexpression in CAFs. GAPDH was used as the loading control. Significance was calculated with Student’s *t* test for (H). ***P < 0.001. (J) The expression of B7-H3, COL1A1, YAP, and phosphor-YAP in control, miR-29a-3p-overexpressed, and B7-H3-rescured CAFs was assessed by Western blotting assay. (K) Subcellular location of YAP1 in control, miR-29a-3p-overexpressed, and B7-H3-rescured CAFs was assessed by immunofluorescence assays. Data are presented as mean ± SD. Significance was calculated using the ANOVA with the Tukey’s multiple-comparison test. ***P < 0.001. (L) The expression of B7-H3 and COL1A1 in control, B7-H3-overexpressed, and Cyto-D-treated CAFs was assessed by Western blotting assay.

To better understand the cellular roles of B7-H3, we investigated the expression of B7-H3 in various cell types. We found that macrophages, cancer-associated fibroblasts (CAFs), and tumor cells were the main cell types that expressed B7-H3 (Fig. 3A). We also validated the expression of B7-H3 in multiple cell lines and found that only Jurkat T cells exhibited low B7-H3 expression (Fig. 3B). We next investigated cellular functions of miR-29a-3p in cell types with B7-H3 high expression. In macrophages, previous studies have reported that B7-H3 could promote M2 polarization via multiple molecular mechanisms [30,31]. As expected, the bioinformatics analysis revealed that B7-H3 expression was associated with decreased M1 signature and increased M2 signature (Fig. 3C). In vitro assays showed that miR-29a-3p could down-regulate B7-H3 expression in macrophages and inhibit M2 polarization (Fig. 3D to F). In addition, overexpression of B7-H3 reversed miR-29a-3p-mediated M2 polarization inhibition (Fig. 3F). The role of B7-H3 in CAFs has not been defined up to date. In CAFs, B7-H3 expression was associated with increased fibrosis activity and miR-29a-3p could down-regulate B7-H3 expression as well as COL1A1 expression (Fig. 3G to J). We next tried to explore the potential transcriptional mechanisms. As previously summarized, nuclear factor κB (NF-κB), Smad 2/3, c-Myb, HIF1α, YAP, and ZEB1 were proved to transcriptionally up-regulated COL1A1 expression [32]. The bioinformatics analysis based on the single-cell RNA sequencing (scRNA-seq) dataset exhibited that Myb and YAP1 were highly expressed in CAFs (Fig. S5A) and only YAP1 was positively correlated with B7-H3 expression (Fig. S5B and C). In vitro assays revealed that miR-29a-3p promoted the phosphorylation of YAP1, inhibited its nuclear translocation, and disturbed the formation of F-actin, and these cellular effects could be reversed by B7-H3 overexpression (Fig. 3J and K). Interestingly, B7-H3 overexpression also promoted COL1A1 expression but could be blocked by cytochalasin D (Cyto-D), the inhibitor for YAP1 by inhibiting actin polymerization (Fig. 3L). Thus, we suspected that miR-29a-3p inhibited type I collagen expression via the B7-H3/F-action/YAP1 axis.

**Fig. 4.**
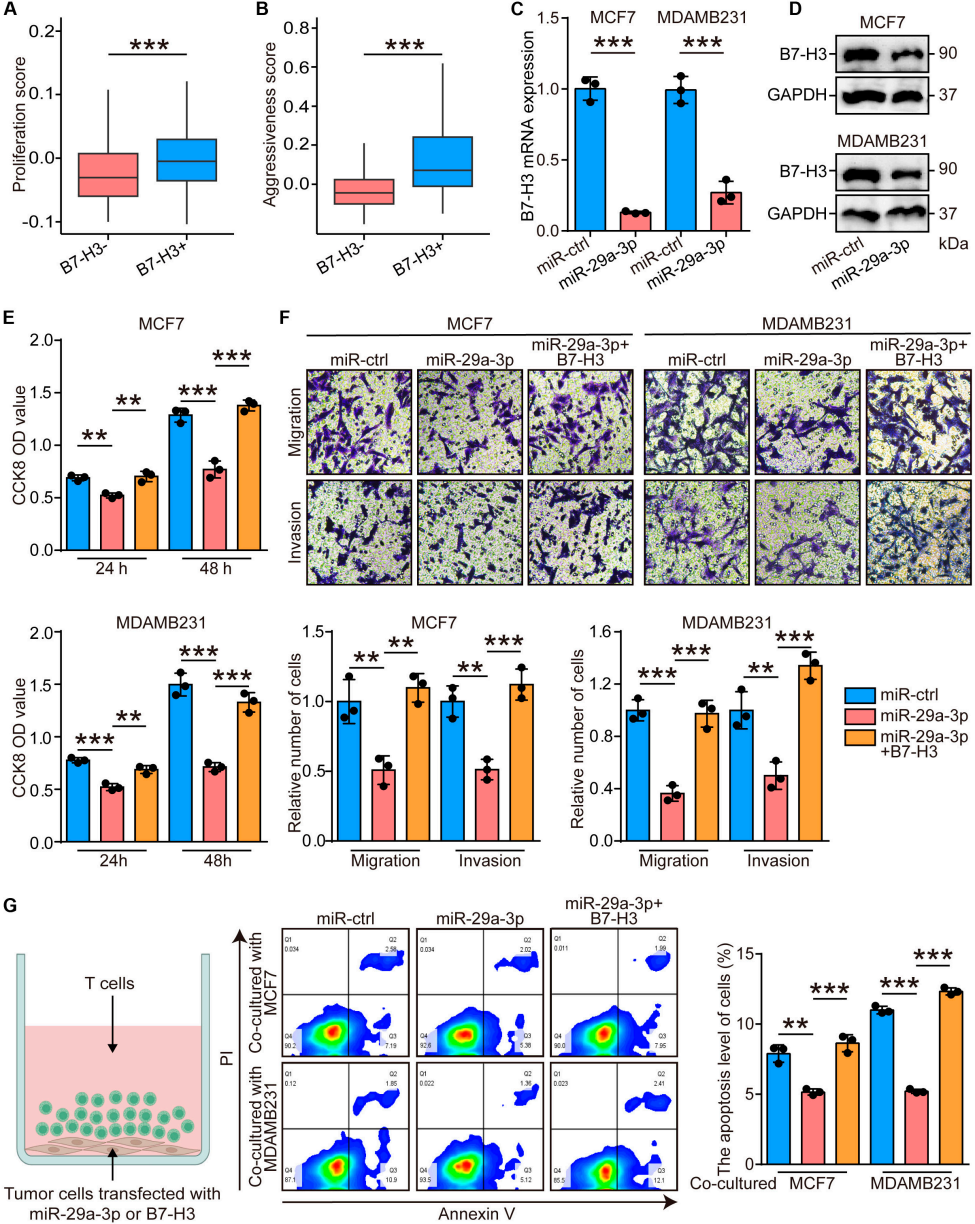
miR-29a-3p inhibited tumor cell aggressiveness and immune escape. (A and B) Level of proliferative and invasive signatures in tumor cells with B7-H3-positive and B7-H3-negative expression. Significance was calculated with Student’s *t* test. ***P < 0.001. (C and D) Down-regulated B7-H3 in both mRNA and protein levels after miR-29a-3p overexpression in MCF7 and MDAMB231 cells. GAPDH was used as the loading control. Significance was calculated with Student’s *t* test for (B). ***P < 0.001. (E) The proliferative capacity of control, miR-29a-3p-overexpressed, and B7-H3-rescured tumor cells was examined at 24 and 48 h after transfection by CCK-8 assay. Data are presented as mean ± SD. Significance was calculated with Student’s *t* test. **P < 0.01, ***P < 0.001. (F) The migratory and invasive capacities of control, miR-29a-3p-overexpressed, and B7-H3-rescured tumor cells were examined at 24 h after transfection by Boyden chamber assay. Total original magnification, 200×. Significance was calculated with Student’s *t* test. **P < 0.01, ***P < 0.001. (G) Apoptosis levels of T cells cocultured with control, miR-29a-3p-overexpressed, and B7-H3-rescured tumor cells were checked by flow cytometry. Data are presented as mean ± SD. Significance was calculated with Student’s *t* test. *P < 0.05, **P < 0.01, ***P < 0.001.

In addition, the cellular functions of miR-29a-3p in tumor cells were also explored. B7-H3 expression was positively related to increased proliferative and invasive activities (Fig. 4A and B). The mRNA and protein level of B7-H3 was remarkably decreased in miR-29a-3p-overexpressed MCF7 and MDAMB231 cells (Fig. 4C and D). Compared with the control cells, MCF7 and MDAMB231 cells with miR-29a-3p overexpression exhibited attenuated proliferative, migratory, and invasive capacities (Fig. 4E and F). Given the role of B7-H3 as an immune checkpoint, we also checked the effects of miR-29a-3p on cytotoxic T cell-mediated antitumor activity. The results showed that tumor cells transfected with miR-29a-3p caused low exhausted levels of cytotoxic T cells (Fig. 4G). Taken together, miR-29a-3p remarkably suppressed cancer progression by regulating macrophages and fibroblast activities and directly inhibiting tumor cells, which could be a novel therapeutic target.

### miR-29a-3p was related to the immuno-hot TME features and decreased collagen deposition

**Fig. 5.**
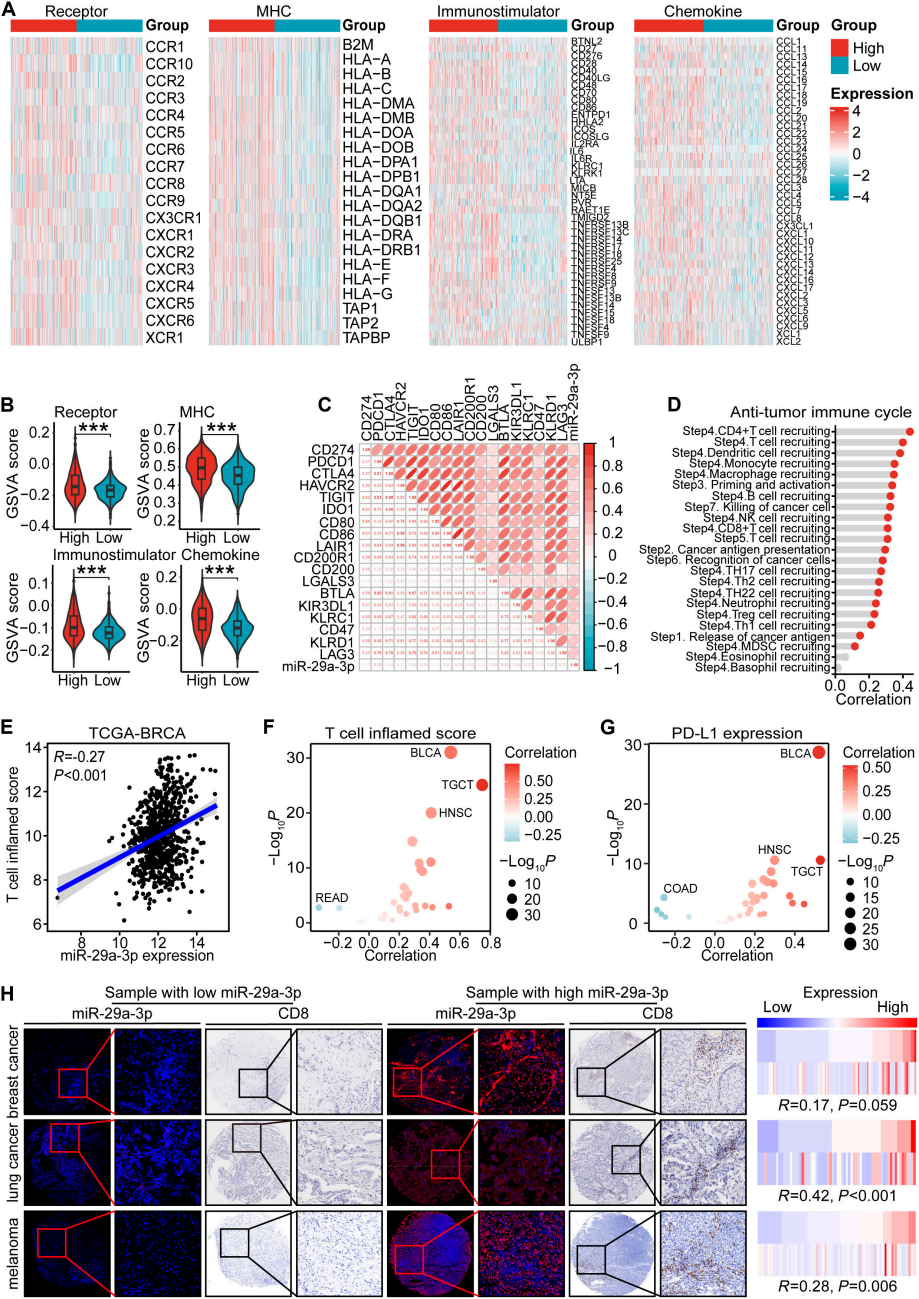
miR-29a-3p was associated with the inflamed TME. (A and B) Expression levels of immunomodulators (MHC, receptors, immunostimulators, and chemokines) in the high- and low-miR-29a-3p groups in BRCA. Data are presented as mean ± SD. Significance was calculated with Student’s *t* test. ***P < 0.001. (C) Correlations between miR-29a-3p and inhibitory immune checkpoints. The color and the values indicate the Pearson correlation coefficient. (D) Correlations between miR-29a-3p and the activities of the various steps of the cancer immunity cycle calculated by ssGSEA algorithm. Significance was calculated with Pearson test. (E and F) Correlation between miR-29a-3p and T cell inflamed score in BRCA and pan-cancer. Data were obtained from the TCGA database. Significance was calculated with Pearson test. (G) Correlation between miR-29a-3p and PD-L1 expression in pan-cancer. Data were obtained from the TCGA database. Significance was calculated with Pearson test. (H) Representative images showing CD8 expression in tumor tissues with low- and high-miR-29a-3p expression in BRCA, lung cancer, and melanoma, along with semiquantitative analysis. Staining data of miR-29a-3p and CD8 in lung cancer and melanoma from our previous study [42] were used as controls. Total original magnification, 50× (left) and 200× (right). Significance was calculated with Spearman test.

Given that miR-29a-3p directly targeted B7-H3, we subsequently explored the immunological role of miR-29a-3p in BRCA and other cancer types using the TCGA cohort. Many chemokines, paired receptors, major histocompatibility complex (MHC) molecules, and immunomodulators were up-regulated in the high-miR-29a-3p group in BRCA (Fig. 5A and B). These chemokines and receptors attract effector TIICs, such as CD8^+^ T cells, macrophages, and antigen-presenting cells. Subsequently, we estimated the infiltration levels of TIICs using the EPIC and TISDB algorithms. We found that the expression of immune cell markers and infiltration levels of most immune cells were remarkably up-regulated in the high-miR-29a-3p group (Fig. S6A to C). It was discovered that inhibitory immune checkpoints, like PD-1/PD-L1, exhibited high expression levels in the inflamed TME [33]. Not surprisingly, miR-29a-3p showed strong correlations with several immune checkpoints in BRCA, including CD274, PDCD1, and CTLA4 (Fig. 5C). The cancer immunity cycle is driven by coordinated actions of chemokines and various immunomodulators. Interestingly, miR-29a-3p was found to have a positive correlation with the activities of the majority of steps within this cycle (Fig. 5D). In addition, miR-29a-3p was also positively correlated with T cell inflamed score in BRCA (Fig. 5E). Further pan-cancer analysis revealed that miR-29a-3p was positively correlated with T cell inflamed score and immune checkpoint expression in most cancer types (Fig. 5F and G and Fig. S7A to D). We also validated the correlation between miR-29a-3p and CD8 level, and found that miR-29a-3p was positively correlated with CD8^+^ T cell level in BRCA, lung cancer, and melanoma (Fig. 5H).

**Fig. 6.**
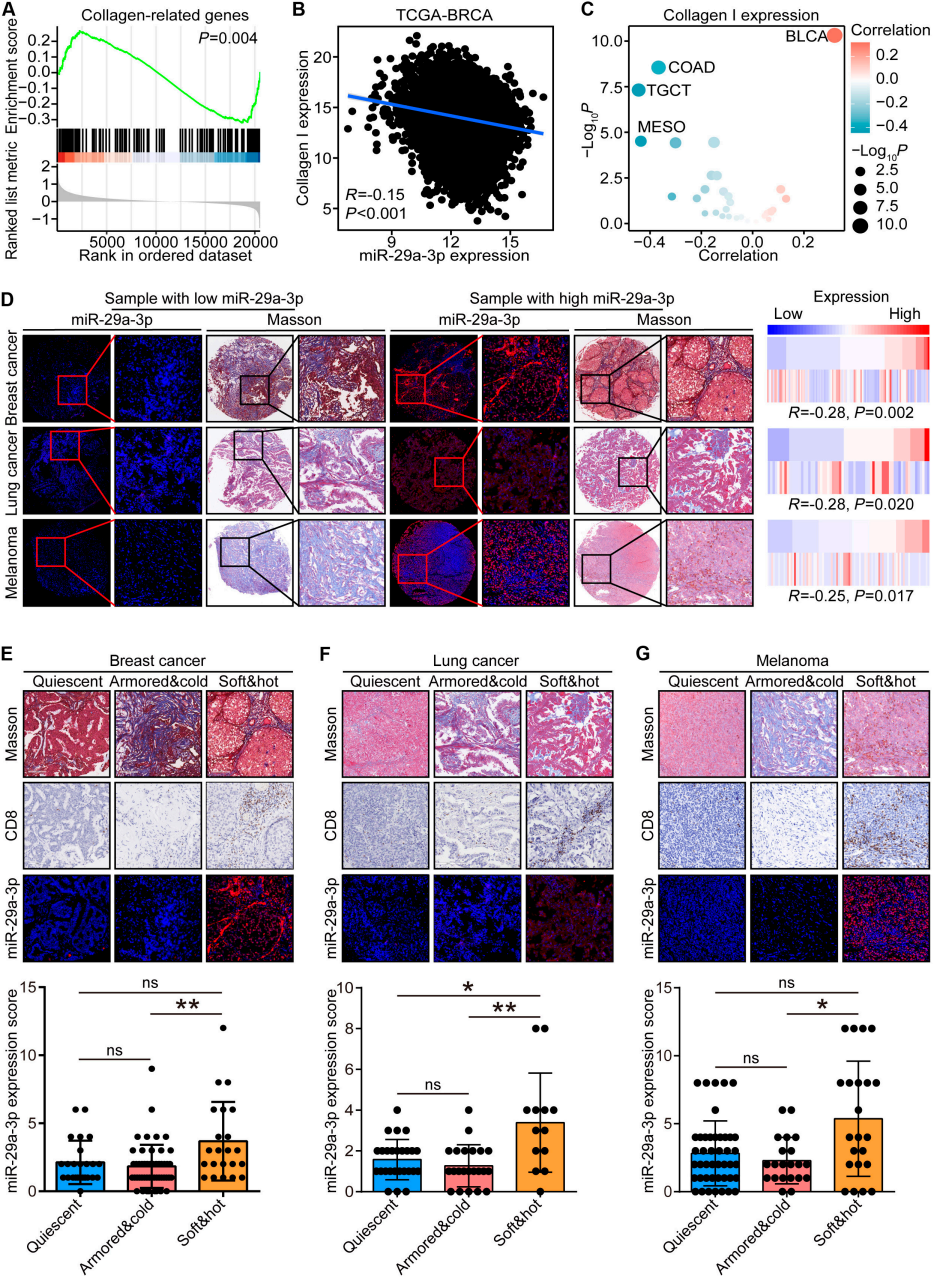
miR-29a-3p was associated with decreased collagen deposition. (A) Gene set enrichment analysis (GSEA) of collagen-related genes in BRCA samples with low- and high-miR-29a-3p expression. (B and C) Correlation between miR-29a-3p and collagen I expression in BRCA and pan-cancer. Collagen I expression was calculated from the mean values of COL1A1 and COL1A2. Data were obtained from the TCGA database. Significance was calculated with Spearman test. (D) Representative images showing collagen area determined by Masson staining in tumor tissues with low- and high-miR-29a-3p expression in BRCA, lung cancer, and melanoma, along with semiquantitative analysis. Staining data of miR-29a-3p and Masson in lung cancer and melanoma from our previous study [42] were used as controls. Total original magnification, 50× (left) and 200× (right). Significance was calculated with Pearson test. (E to G) Representative images uncovering miR-29a-3p expression in samples with different subtypes in BRCA, lung cancer, and melanoma and semiquantitative analysis. Data are presented as mean ± SD. Significance was calculated with Kruskal–Wallis with Dunn’s multiple-comparison test. *P < 0.05, **P < 0.01.

As previously described, genes up-regulated in the low miR-29a-3p group were mostly enriched in the “extracellular matrix organization” term and the negative regulation of miR-29a-3p on CAFs. We assessed correlations between miR-29a-3p and extracellular matrix (ECM) factors using public and in-house clinical cohorts, and the results showed that miR-29a-3p was negatively related to most collagens in the TCGA-BRCA cohort (Fig. 6A). In addition, we found that miR-29a-3p was negatively correlated with type I collagen, the most abundant collagen subtype, in not only BRCA but also most solid cancer types (Fig. 6Fig. 6B and C). Further validation revealed that miR-29a-3p was negatively correlated with the collagen level in BRCA, lung cancer, and melanoma (Fig. 6D). In addition, as we previously reported [34], armored and cold tumor is one subtype with poor prognosis and resistance to immune checkpoint blockade. Given the positive correlation with immune infiltration and negative correlation with collagen deposition of miR-29a-3p, we examined the expression of miR-29a-3p across various immuno-collagenic subtypes. The results showed that miR-29a-3p was the lowest in armored and cold tumors in BRCA, lung cancer, and melanoma (Fig. 6E to G). Taken together, miR-29a-3p was negatively correlated with collagen deposition and decreased in armored and cold subtypes. In summary, miR-29a-3p was highly correlated with the inflamed TME.

### Liposome-encapsulated miR-29a-3p activated TME and boosted anti-B7-H3 therapy

**Fig. 7.**
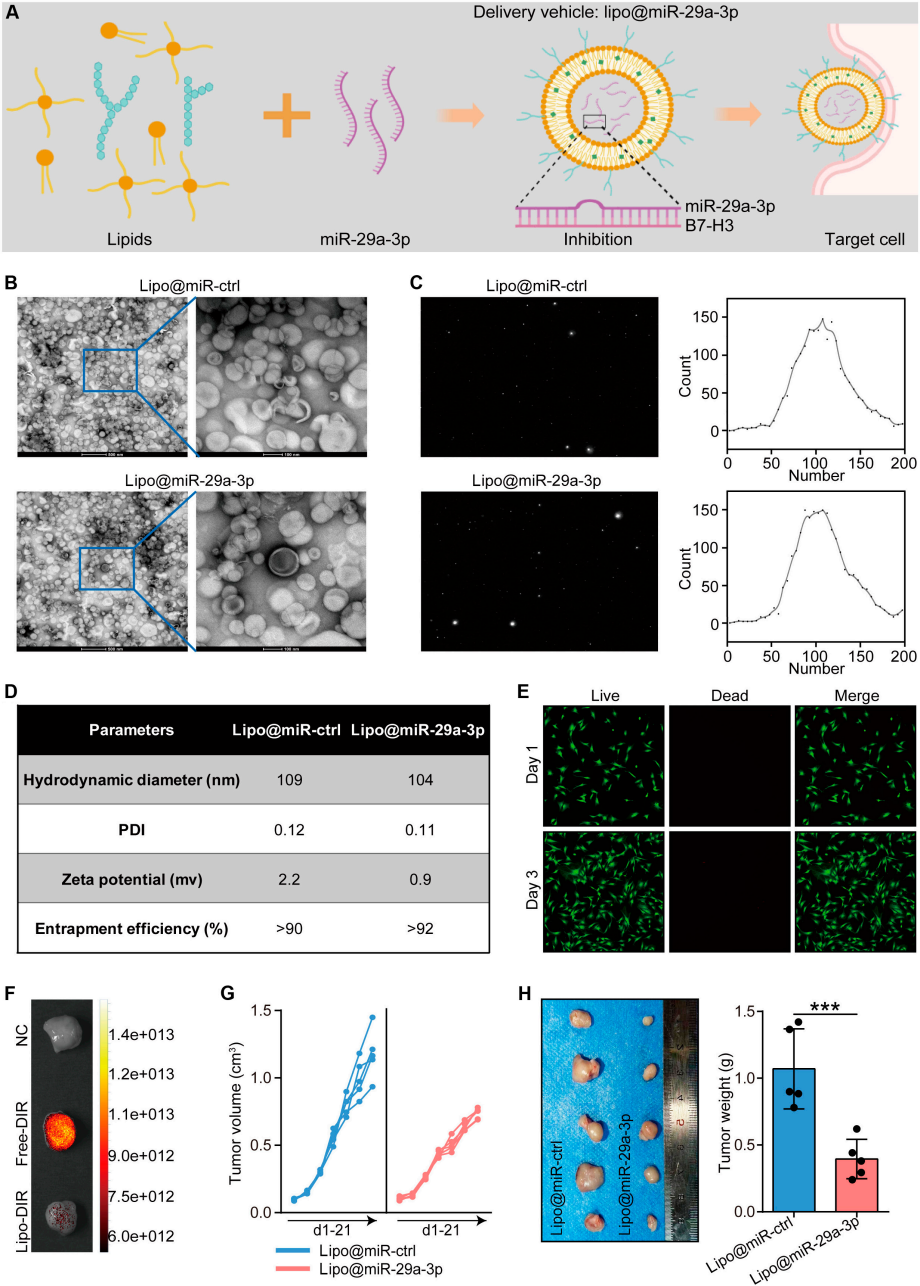
Characterization and antitumor effects of Lipo@miR-29a-3p. (A) Diagram of the synthesis steps of lipo@miR-29a-3p. (B) Transmission electron micrographs of lipo@miR-29a-3p and lipo@miR-ctrl. Scale bar, 500 or 100 nm. (C) NTA characterization plots of the liposome. The plot on the left shows the microscopic size of the liposome, and the plot on the right is a graph of the data from the particle size analysis. (D) Table of parameters for hydrodynamic diameter, polydispersity, zeta potential, and stability. (E) Cell viability of primary stem cells from the Achilles tendon of mice cocultured with the liposomes for 1 and 3 d was evaluated using the live/dead staining. Total original magnification, 200×. (F) Fluorescence tracing of free DIR dye or DIR-lipo@miR-29a-3p or NC-lipo@miR-29a-3p for tumors. (G) Effect of lipo@miR-29a-3p on tumor volume in BALB/C mice bearing 4T1 cells. (H) Effect of lipo@miR-29a-3p on tumor weight in BALB/C mice bearing 4T1 cells and quantitative analysis. Data are presented as mean ± SD. Significance was calculated with Student’s *t* test. ***P < 0.001.

We have made initial efforts to clinically apply the findings from this study. Liposomes with a unique structure were employed to package miR-29a-3p (Fig. 7A). Transmission electron microscopy (TEM) results showed that lipo@miR-ctrl and lipo@miR-29a-3p possessed similar shapes (i.e., elliptical), and their numbers and particle sizes were basically the same, with a particle size of roughly 100 nm (Fig. 7B). Nanoparticle tracking analysis (NTA) images and parametric analyses confirmed that the particle sizes of the 2 liposomes were distributed in the interval of 50 to 200 nm and were mainly concentrated at around 100 nm (Fig. 7C). Other parameters such as hydrodynamic diameter, polydispersity, zeta potential, and stability were assessed in Fig. 7D, and there was no significant difference between the 2 liposomes in these parameters. At the cellular level, cell viability staining showed that lipo@miR-ctrl did not promote stem cell death at 1- and 3-d treatment times (Fig. 7E). In vitro release experiments showed that lipo@miR-29a-3p exhibited slow and stable miRNA release under specific conditions and miRNA release from this liposomal system was stable at different temperatures (Fig. S8A and B). In addition, the expression of miR-29a-3p in tumor tissues from mouse receiving lipo@miR-29a-3p therapy was significantly up-regulated (Fig. S8C). All these results demonstrated the good targeting efficiency of this material system. In animals, staining of tissue sections suggested that lipo@miR-ctrl and lipo@miR-29a-3p did not cause structural damage or immune cell infiltration in the heart, liver, spleen, lung, and kidney, suggesting that the biomaterial is biocompatible (Fig. S9A and B). Further results showed that there were no significant differences in these serum markers between the liposome groups (empty and miR-29a-3p-loaded) and the control group, indicating that the liposomal formulations exhibit good biocompatibility and do not induce apparent liver or kidney toxicity (Fig. S10A to D). More importantly, 1,1-dioctadecyl-3,3,3,3-tetramethylindotricarbocyanine iodide (DIR)-tracing results showed that lipo@miR-29a-3p was present in tumor tissues for at least 3 d, revealing that lipo@miR-29a-3p could reach tumor tissues (Fig. 7F). In the mouse tumor model, intravenous injections of lipo@miR-29a-3p significantly inhibited tumor growth but did not change the mouse weight (Fig. 7G and H and Fig. S9C), exhibiting encouraging antitumor activity.

**Fig. 8.**
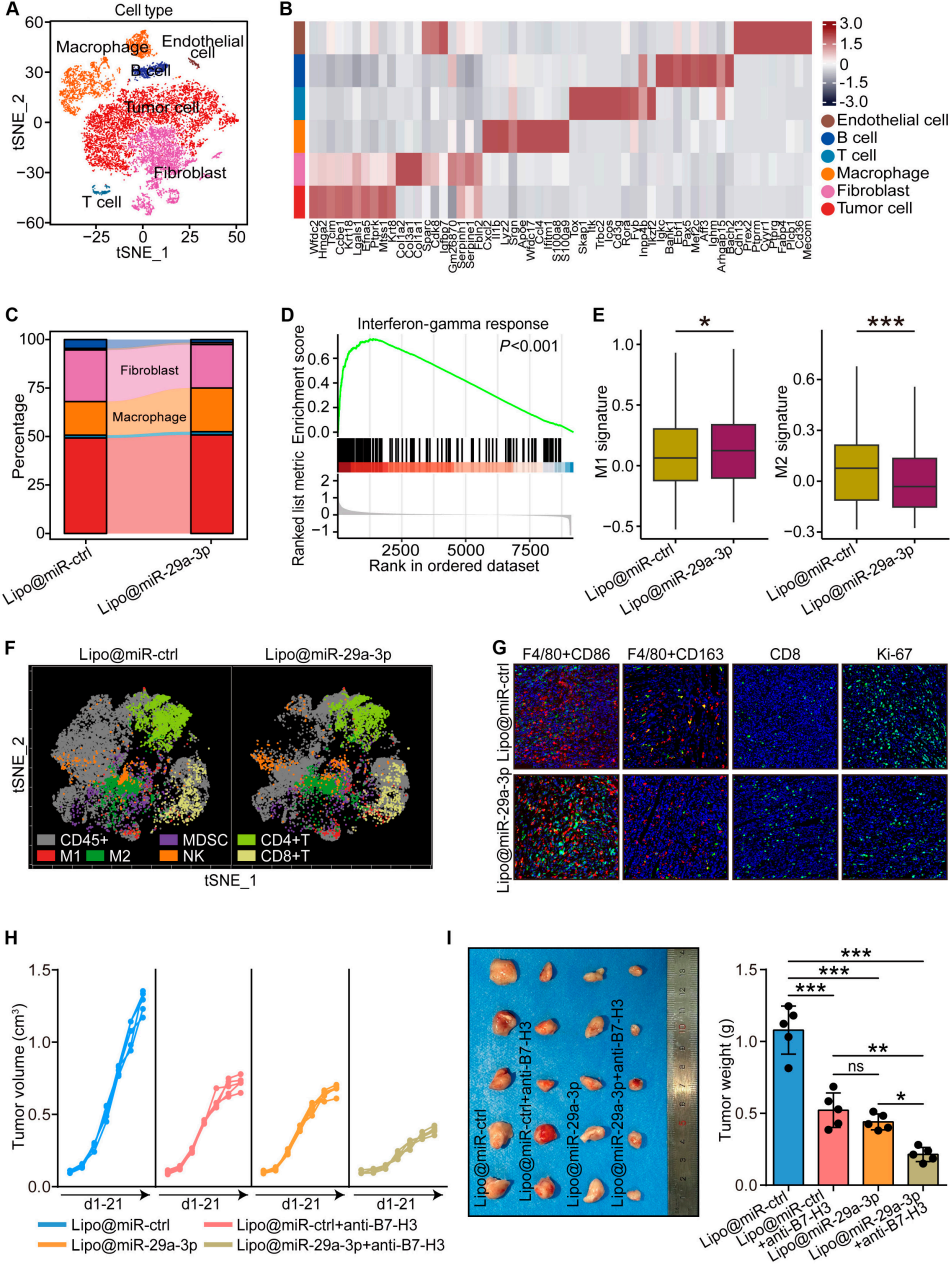
Lipo@miR-29a-3p activated TME and boosted anti-B7-H3 therapy. (A) t-SNE visualization of single cells passed quality controls with different cell subtypes. (B) Heatmap for gene expression levels of top 10 cell type-specific genes. (C) Difference in cell subtypes in lipo@miR-ctrl- and lipo@miR-29a-3p-treated tumor tissues. (D) GESA of interferon-γ response in macrophages from tumor tissues with lipo@miR-ctrl and lipo@miR-29a-3p treatment. (E) Level of M1 and M2 signature in macrophages from tumor tissues with lipo@miR-ctrl and lipo@miR-29a-3p treatment. The scores were calculated by “AddModuleScore” function in the Seurat package. Significance was calculated with Student’s *t* test. *P < 0.05, ***P < 0.001. (F) CyTOF showed the typic trend of each cellular type in the TME. (G) The expression of M1 and M2 macrophage markers, CD8, and Ki-67 was examined by immunofluorescence assay. Total original magnification, 200×. (H) Effect of B7-H3 monoclonal antibody (mAb), lipo@miR-29a-3p, and combination on tumor volume in BALB/C mice bearing 4T1 cells. (I) Effect of B7-H3 mAb, lipo@miR-29a-3p, and combination on tumor weight in BALB/C mice bearing 4T1 cells and quantitative analysis. Data are presented as mean ± SD. Significance was calculated with one-way ANOVA with Tukey’s multiple-comparison test. *P < 0.05, **P < 0.01, ***P < 0.001.

We next examined the effects of lipo@miR-29a-3p on the TME using state-of-the-art techniques including the scRNA-seq and the cytometry by time-of-flight (CyTOF) technologies. Compared with control groups, the scRNA-seq analysis revealed that lipo@miR-29a-3p notably increased macrophage number and decreased fibroblast number (Fig. 8A to C). Given the significant effects of miR-29a-3p on macrophages in vitro, we further validated the in vivo effects of miR-29a-3p. Compared with control groups, lipo@miR-29a-3p activated the interferon-γ response in macrophages, promoted M1 polarization, and inhibited M2 polarization (Fig. 8D and E). To further provide a more holistic view of the TME changes induced by miR-29a-3p, we perform a high-resolution dissection of interactions among various cell types in the microenvironment of the control and lipo@miR-29a-3p groups based on the combined expression of multi-subunit ligand–receptor complexes. The number of interactions among different subpopulations was compared between the control and lipo@miR-29a-3p groups. Results showed that the immune cell subpopulations in the lipo@miR-29a-3p group presented significantly more interactions than those from the control group (Fig. S11A), especially between the macrophages and T cells (Fig. S11B). Macrophages communicated with T cells via CD40-CD40LG, CD28-CD80, and CD28-CD86 (Fig. S11C), which have been reported to mediate the potent T cell-stimulatory and antitumor activity [35–37], indicating that lipo@miR-29a-3p therapy could alter the immunosuppressive TME to the immunoactivated status to enhance the antitumor immunity. In addition, lipo@miR-29a-3p inhibited the activation of CAFs, and Masson staining also showed decreased collagen deposition in lipo@miR-29a-3p-treated tumors (Fig. S12A and B). CyTOF was also used to further analyze the TME landscape. Similar findings that M1 macrophages and CD8^+^ T cells were increased and M2 macrophages were decreased were observed (Fig. 7F and Fig. S13A). Furthermore, immunofluorescence and flow cytometry were used to validate these results (Fig. 8G and Fig. S13B and C). Also, lipo@miR-29a-3p inhibited B7-H3 and Ki-67 expression (8G and Fig. S13D). In addition, CyTOF and flow cytometry analyses uncovered that lipo@miR-29a-3p inhibited CD8^+^ T cell exhaustion, which contributed to the inflamed TME (Fig. S13E and F).

B7-H3 has been identified as a novel therapeutic target in armored and cold tumors in our previous research [34,38]. Given that miR-29a-3p directly targeted B7-H3 and the activated effects on the TME, we suspected that miR-29a-3p could enhance anti-B7-H3 therapy. Compared with the previously shown lipo@miR-29a-3p-treated BRCA-bearing mice (Fig. 7G and H), anti-B7-H3 showed similar antitumor effect of lipo@miR-29a-3p (Fig. 8H and I). Surprisingly, the combination of lipo@miR-29a-3p and anti-B7-H3 resulted in a significant improvement (8H and I), but the weight of mouse did not change (Fig. S9D), suggesting the supportive effect of miR-29a-3p on anti-B7-H3 therapy and controlled tolerance. All these results demonstrated that lipo@miR-29a-3p attacked armored and cold tumors and boosted anti-B7-H3 therapy.

## Discussion

Typically regarded as a tumor suppressive gene, miR-29a-3p is a small RNA that exerts a critical role in tumors [39]. Its function primarily involves the regulation of multiple target genes to alter oncogenesis and tumor progression. In tumor tissues, the expression levels of miR-29a-3p are usually down-regulated, which may lead to a series of biological effects, including promoting tumor cell proliferation, migration, and invasion, while inhibiting cell apoptosis [39–41]. Notably, several collagens, including COL1A1, COL3A1, COL4A1, and COL5A1, have been identified as the targets of miR-29a-3p [42–44]. Therefore, miR-29a-3p could be used as a therapeutic target for cancer treatment. As an exercise-responsive miRNA, exercise up-regulates the expression of miR-29a-3p in muscle tissue, and promotes its release into circulation, thereby impacting the function of cells in various tissues, participating in the regulation of multiple biological processes [24,45].

In this research, we performed a systematic analysis of expression levels and prognostic values of exercise-responsive miRNAs by using large-scale RNA-seq data in BRCA and pan-cancer, and found that miR-29a-3p was down-regulated in most cancer types and associated with poor prognosis. More importantly, tumoral miR-29a-3p expression was positively related to immunomodulators and TIIC levels across cancers. Theoretically, given that miR-29a-3p is a key player in responding to inflammation to a certain degree, it is possible that miR-29a-3p could have a substantial impact on tumor immunity regulation. However, its precise role as an immunomodulator in human cancers remains unclear.

The regulation of antitumor immune status in the TME by exercise is a highly researched area of interest [15]. Exercise can affect the tumor immune microenvironment in various manners, including regulating the number, activity, and function of immune cells, influencing the interaction between immune cells and tumor cells, and thereby impacting the growth, metastasis, and treatment of tumors [15]. It has been reported that exercise contributes to tumor management by enhancing CD8^+^ T cell infiltration via mediating the CXCR3 signaling and thus boosts the response to immune checkpoint blockade in BRCA [46]. In addition, our previous study found that squalene epoxidase, an essential gene to regulate immune escape in tumor cells by modulating cholesterol biosynthesis [47,48], could be down-regulated by physical exercise [14]. Overall, exercise contributes to enhancing antitumor immunity, but the underlying molecular mechanisms may be complicated and not well understood.

B7-H3 is a newly focused immune checkpoint in cancers [49]. Although its receptor is not yet clear, the high expression of B7-H3 on the surface of tumor cells can inhibit the activation and function of T cells by binding to its potential receptors, thereby inhibiting the antitumor immune response [50]. Furthermore, B7-H3 can accelerate the proliferation and invasion capabilities of tumor cells, directly participating in the regulation of tumor growth and metastasis [51,52]. In addition to tumor cells, previous studies have revealed that B7-H3 promoted M2 polarization via multiple mechanisms in macrophages [30,31].

Recently, multiple clinical trials (such as NCT02923180 and NCT02475213) have confirmed the antitumor effectiveness and safety of enoblituzumab [53], a humanized Fc-engineered B7-H3-targeting antibody, in patients with localized prostate cancer (PCa), advanced head and neck squamous cell carcinoma, and non-small cell lung cancer (NSCLC) [54,55]. Notably, a phase II neoadjuvant PCa trial (NCT02923180) demonstrated that CD8 T cell density in prostatectomy samples was significantly higher in enoblituzumab-treated patients compared with age-matched and stage-matched untreated prostatectomy controls [53], suggesting that targeting B7-H3 could improve the survival by accelerating the infiltration of CD8^+^ T cells to enhance the antitumor response. Among our previously established immuno-collagenic subtypes, B7-H3 was found to be highly expressed in armored and cold tumors, one refractory tumor type that is resistant to immune checkpoint blockade. Combined with the observation in clinical trials, targeting B7-H3 may be a critical therapeutic strategy for refractory tumors.

The expression of B7-H3 can be regulated at multiple levels, including transcription factors and miRNAs. Some transcription factors can directly or indirectly regulate the expression of B7-H3. For example, transcription factors such as NF-κB can bind to the promoter region of B7-H3 and thus promote its transcription [56]. In addition, miRNA is also a significant regulator for B7-H3 expression affecting its stability or translation process [57,58]. In this study, we found that exercise-responsive miR-29-3p could directly bind to the 3′-UTR of B7-H3 and inhibit its expression. As expected, there was a negative correlation observed between miR-29a-3p and the expression of B7-H3 and miR-29a-3p was lowly expressed in armored and cold tumors [34], suggesting that overexpression of miR-29-3p could be used as an effective therapy for refractory armored and cold tumors. What is more, we found that miR-29-3p decreased collagen I expression by inhibiting the B7-H3/F-actin/YAP1 axis in CAFs. To some extent, our findings explained the potential mechanisms of exercise-mediated activation of the tumor immune microenvironment.

We found that miR-29a-3p could inhibit matrix metalloproteinase 2 (MMP2) and MMP9 expression, and overexpression of B7-H3 recovered MMP expression (Fig. S14A). In addition, B7-H3 was also positively correlated with MMP2 and MMP9 in the TCGA-BRCA dataset (Fig. S14B and C). Although miR-29a-3p down-regulated COL1A1, it also decreased MMP expression. Moreover, it has been reported that miR-29a-3p could even inhibit tumor metastasis [59]. Thus, we believe that miR-29a-3p does not cause tumor metastasis.

Although existing evidence suggests that miR-29a-3p can serve as a potential nucleic acid drug for armored and cold tumors, its susceptibility to degradation and poor stability in vivo determine that appropriate carriers for encapsulation and delivery to ensure its effective delivery to target cells are required [60]. Given that miR-29a-3p could be transferred by extracellular vesicle in the physiological condition [42], we employed liposome therapy to model this physiological effect. Liposomes are commonly used nanocarriers for transporting various bioactive substances. Liposomal encapsulation of small RNA therapy offers multiple advantages, such as specificity, stability, and safety, making it a promising approach for treating various diseases [61,62]. We established a novel nucleic acid drug, lipo@miR-29a-3p, and in vivo assays exhibited hypotoxicity. In addition, lipo@miR-29a-3p showed active antitumor activity and activated the immune microenvironment in remodeling the cellular status of macrophages, namely, activating the interferon-γ response in macrophages, promoting M1 polarization, and inhibiting M2 polarization. Macrophages played a crucial role in immune activation. Numerous studies proved the tumorigenicity of M2 macrophages in multiple solid carcinomas by accelerating tumor growth, the formation of immunosuppressive TME, and the acquisition of therapeutic resistance [63], while proinflammatory polarization of M2 macrophages can reactivate the immune microenvironment and enhance the antitumor response [64]. Totally, lipo@miR-29a-3p can transfer the TME from immunosuppressive to immune-activated status, thereby sensitizing anti-B7-H3 therapy.

The effectiveness of lipo@miR-29a-3p in tumor accumulation can be attributed to the enhanced permeability and retention effect commonly observed in tumor vasculature. This phenomenon allows nanoparticles, such as liposomes, to passively accumulate in tumor tissues due to their leaky vasculature and poor lymphatic drainage [65,66]. Further study may utilize the surface modification of liposomes with targeting ligands, which can further increase their accumulation and uptake by tumor cells, promoting the localized release of miR-29a-3p and subsequent therapeutic actions within the TME. The ability of lipo@miR-29a-3p to modulate the immune microenvironment also plays a significant role in its antitumor efficacy. By altering the expression levels of specific miRNAs within tumor cells, lipo@miR-29a-3p can disrupt the immunosuppressive conditions of armored and cold tumors, thus reactivating immune surveillance and response. This dual mechanism of direct antitumor activity combined with immune modulation underscores the potential of lipo@miR-29a-3p as a multifunctional therapeutic agent in oncology.

In translating our findings into clinical applications, several critical aspects must be considered, including potential side effects, delivery methods, and the long-term efficacy of miR-29a-3p therapy. Our in vivo studies demonstrated that lipo@miR-29a-3p exhibited excellent biocompatibility, as evidenced by histopathological analyses of major organs (heart, liver, spleen, lungs, and kidneys), which showed no significant tissue damage or immune cell infiltration. However, we acknowledge that long-term safety remains a concern, and future studies will involve larger cohorts with extended follow-up periods to rigorously assess any delayed adverse effects. In terms of delivery, liposomes were chosen due to their high encapsulation efficiency, stability, and ability to target tumor tissues effectively. Our fluorescence tracking experiments confirmed that lipo@miR-29a-3p accumulated predominantly in tumor tissues with minimal off-target distribution, underscoring its potential as a targeted delivery system. Nevertheless, we are also exploring alternative delivery platforms, such as exosome-based or nanoparticle-based carriers, to further enhance the specificity and efficiency of miRNA delivery in clinical settings. Finally, while our current study highlights the short-term antitumor effects of lipo@miR-29a-3p, assessing its long-term therapeutic efficacy is paramount. Future research will focus on long-term studies in preclinical models to evaluate sustained tumor suppression, potential resistance mechanisms, and therapeutic durability. These steps are essential for advancing miR-29a-3p-based therapies toward clinical trials.

### Conclusion

**Fig. 9.**
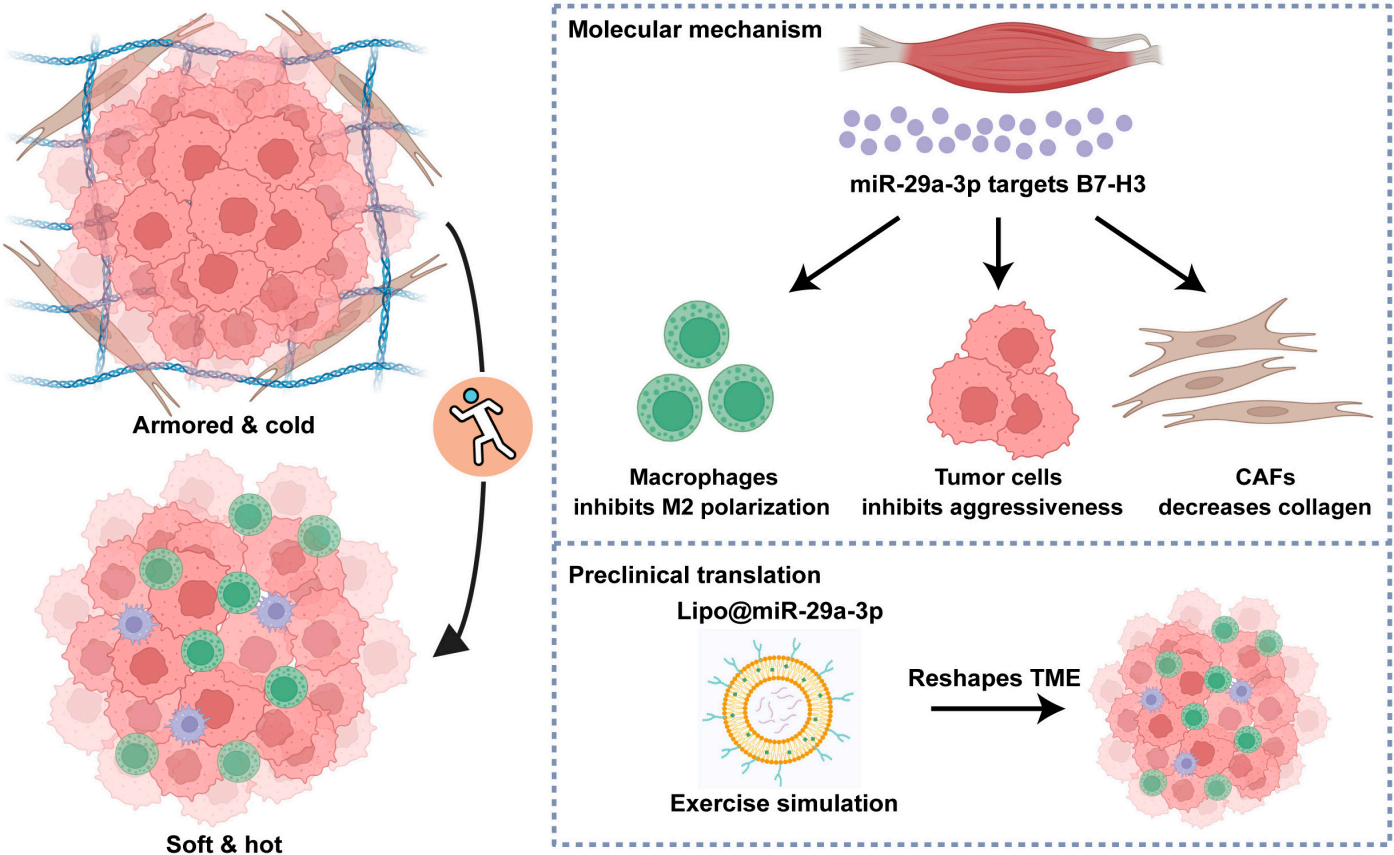
Schematic overview of the current study. Exercise-responsive miR-29a-3p can make armored and cold tumors turn into soft and hot tumors, which greatly increases its therapeutic significance in clinical practice. Mechanistically, miR-29a-3p targets B7-H3 expression to inhibit tumor cell aggressiveness, macrophage M2 polarization, and collagen synthesis of fibroblasts. Translationally, a novel biomaterial lipo@miR-29a-3p was developed to simulate the benefits of exercise. Copyrighted from the BioRender platform.

To sum up, we identified miR-29a-3p as a significant exercise-responsive miRNA, which attacked armored and cold tumors by inhibiting B7-H3 expression. In addition, miR-29a-3p could be used as a nucleic acid drug and lipo@miR-29a-3p could be a promising antitumor medicament for refractory armored and cold tumors (Fig. 9). Overall, we reveal the mechanisms underlying the antitumor effects of exercise and provide miR-29a-3p restoration as an alternative strategy for antitumor therapy.

### Limitations of the study

We acknowledge certain limitations of our study. The first relates to the source of miR-29a-3p. Although multiple studies confirmed the up-regulation of miR-29a in muscle by exercise [67–69], how exercise affects miRNA transcription and how the miR-29a-3p regulation is induced in the different cell types are still largely unknown. Second, miRNAs are known as the multi-target functions. Whether miR-29a-3p decreases other target expression to trigger similar biological effects is undiscovered. In addition, there will be potential off-target effects due to our material system: (a) liposome surface properties: the surface charge, hydrophilicity, and other properties of liposomes may affect their interactions with cell membranes, thus increasing the risk of off-target binding; (b) miRNA sequence specificity: although miRNAs have a high degree of specificity, they may still be able to nonspecifically bind with other nontargeted RNA sequences in complex biological environments; (c) TME complexity: there are a large number of cell types, growth factors, and signaling molecules in the TME, which may affect the distribution and mechanism of action of liposomes and miRNAs, leading to off-target effects. Follow-up studies should be conducted to optimize liposome design, screen highly specific miRNA sequences, use other therapeutic means in combination, and strengthen monitoring and evaluation, which can effectively reduce the occurrence of off-target effects and improve the safety and efficacy of treatment. We acknowledge the importance of the targets of exercise-derived miR-29a-3p in explaining various exercise-related benefits, which warrants further investigation.

## Methods

### Acquisition of public dataset

Transcriptome profiles, clinical information, and genomic alterations of pan-cancer in TCGA datasets were downloaded from the University of California Santa Cruz (UCSC) Xena (https://xenabrowser.net/datapages/). Quantification data of stem-loop miRNA and miRNA mature strand were also downloaded from the USUC Xena. Samples with both transcriptome and miRNA profiles were selected for further analysis. The scRNA-seq data of primary BRCA tissues were described in our previous research [70].

### Definition of exercise-responsive miRNAs

According to a published systematic review [24], a total of 25 miRNAs were categorized as exercise-responsive as more than 70% of studies suggested concordance in up-regulation in blood, including miR-132, miR-142, miR-143, miR-145, miR-150, miR-155, miR-15a, miR-181a, miR-181b, miR-206, miR-21, miR-222, miR-223, miR-24, miR-29a, miR-29b, miR-29c, miR-30a, miR-30e, miR-338, miR-34a, miR-424, miR-451a, miR-532, and miR-7. The differential expression of these miRNAs between para-tumor and tumor tissues was checked using the stem-loop miRNA expression data obtained from the TCGA-BRCA dataset, and the prognostic values of these miRNAs were explored using the Kaplan–Meier Plotter tool [71].

### Functional enrichment analysis

Based on the median value of miR-29a-3p, we divided the patients in the TCGA-BRCA cohort into miR-29a-3p-high and miR-29a-3p-low groups. Then, the R package “limma” [72] was used to identify the DEGs for miR-29a-3p-high and miR-29a-3p-low groups, respectively. Genes with fold change (FC) ≥ 1.5 and adjusted *P* value < 0.05 were defined as DEGs for the miR-29a-3p-high group, while those with FC ≤ −1.5 and adjusted *P* value < 0.05 were recognized as the DEGs for the miR-29a-3p-low group. To further describe the biological progresses of DEGs up-regulated in the miR-29a-3p-high group, the enrichment analysis was performed by the “GSEA” function in the “clusterProfiler” package [73] in terms of gene signatures from a previous study [74]. In addition, signatures for collagen-related genes and interferon-γ response were obtained from our previous research [34] and the Hallmark dataset, respectively.

### Target gene prediction and dual-luciferase activity assay

The target genes of miR-29a-3p were predicted using TargetScan [75] and miRDB [76] tools. In addition, to intersect believable immune-related target genes of miR-29a-3p, genes negatively correlated with B7-H3 in the TCGA-BRCA dataset (Pearson *R* ≤ −0.2) and the list of immune-related genes [33] were used as qualifications. A WT 3′-UTR fragment and a binding site mutated 3′-UTR fragment of B7-H3 were synthesized for dual-luciferase activity assay [77].

### Bioinformatics analysis of the TME features

The TME features encompassed immunomodulators, the dynamics of the cancer immunity cycle, levels of TIICs, and the presence of inhibitory immune checkpoints. The TCGA dataset was utilized to explore the correlations between miR-29a-3p and these TME features. The detailed description could be found in our previous study [33].

### Collection of clinical samples

Paraffin-embedded human cancer or para-tumor tissue microarrays (TMAs) (HBreD140Su03, HLugA150CS01, HLugS020PG01, HMelC112CD01, and HBreD077Su01) were obtained from the National Engineering Center for Biochip (Shanghai, Outdo Biotech). Comprehensive clinicopathological characteristics and follow-up information were acquired from Outdo BioTech. The Clinical Research Ethics Committee at Outdo Biotech approved the ethical use of these TMAs.

In addition, paired serum and tumor samples from a total of 32 BRCA patients with or without exercise habits from 2020 to 2023 were recruited by Wuxi Maternal and Child Health Care Hospital. These patients were divided into exercise or non-exercise groups according to their leisure-time physical activity (LTPA). LTPA was performed at each patient’s discretion, and the activity intensity was 3 or more metabolic equivalents (METs) according to physical activity guidelines [9]. Physical activity of patients was assessed by asking about discrete activities like walking, running, or swimming, or, alternately, by inquiring about overall weekly participation in moderate to vigorous intensity activities. Samples were obtained before the start of any antitumor therapy. The Clinical Research Ethics Committees at Wuxi Maternal and Child Health Care Hospital approved the recruitment. Detailed information of in-house and public cohorts can be found in Table S1.

### Human cancer tissue staining and quantification of staining results

Fluorescence in situ hybridization (FISH), immunohistochemistry (IHC) staining, Masson staining, and hematoxylin and eosin (H&E) staining were conducted on the above TMAs and tissue slides. For FISH, a signal amplification by exchange reaction-based method was applied (Table S2) [78]. Briefly, tissue sections or TMAs were digested with proteinase K and treated with acetic anhydride. Samples were then incubated in a pre-hybridization solution (catalog G3016-4, Servicebio) for 30 min at 43 °C. Each sample was incubated with 1 μg of probe in 120 μl of phosphate-buffered saline (PBS) overnight. Samples were then incubated with digoxin (DIG)-labeled probes for signal amplification of the hybridization solution (catalog G3016-3, Servicebio) for 3 h at 37 °C. After thorough washing, samples were incubated with a horseradish peroxidase-conjugated anti-DIG antibody for 50 min at 37 °C. For detection, CY3-tyramide was added on the slides for 5 min. Signals were detected using a confocal microscope. Standard operating procedures were followed for IHC and H&E staining. The primary antibodies applied in the research were as follows: anti-CD8 (ready-to-use, catalog PA067, Abcarta) and anti-B7-H3 (1:20,000 dilution, catalog ab219648, Abcam). Antibody staining was visualized with DAB and hematoxylin counterstain. Masson staining was performed on TMAs and tissue slides to determine the collagen deposition using the trichrome stain (Masson) kit (catalog FH115100, FreeThinking, Nanjing, China) according to the manufacturer’s instructions.

The assessment methods for TIIC score, Masson area, B7-H3 combined positive score (CPS), and immuno-collagenic subtypes were described in the previous study [34]. The expression of miR-29a-3p was evaluated by examining the mean fluorescence intensity using ImageJ. In addition, CD8^+^ T cell was counted as a number per mm^2^ by a senior pathologist.

### Reagents for in vitro assays

The inhibitor for actin polymerization and nuclear translocation of YAP1 and Cyto-D (catalog HY-N6682) was purchased from MedChemExpress (Shanghai, China). Tetramethyl rhodamine isothiocyanate (TRITC) phalloidin (catalog 40734ES75) was obtained from Yeasen (Shanghai, China). ImmunoCult human CD3/CD28 T cell activator (catalog 10971) was purchased from STEMCELL Technologies (Vancouver, Canada). The B7-H3 overexpression lentivirus vector was constructed by Genechem (Shanghai, China).

### Cell lines and cell culture

Primary CAFs were isolated from breast tumor tissues [79] and cultured using a primary cell culture medium (catalog CX0013, Yuchi, Shanghai, China). Written consent was obtained from patients, and ethical approval was granted by The Clinical Research Ethics Committees at Wuxi Maternal and Child Health Care Hospital. Human cancer cell lines MDAMB231 (catalog KGG3220-1), MCF7 (catalog KGG3332-1), mouse cancer cell line 4T1 (catalog KGG2224-1), and HEK293T (catalog KGG3108-1) were purchased from KeyGEN (Nanjing, China). THP1 mononuclear cell line (catalog SC0071) was purchased from Yuchicell (Shanghai, China). MDAMB231 cells were cultured in the L15 medium; MCF7, 4T1, and HEK293T cells were cultured in the Dulbecco’s modified Eagle’s medium (DMEM); and THP1 cells were cultured in the RPMI 1640 medium with 0.05 mM 2-thioethanol. All media were added with 10% fetal bovine serum (FBS) at 37 °C with or without 5% CO_2_. All human cell lines were authenticated using short tandem repeat profiling, and all assays were conducted with mycoplasma-free condition. For conversion of THP1 cells from mononuclear cells into macrophages, 200 ng/ml phorbol 12-myristate 13-acetate (PMA; catalog HY-18739) was added to the medium. We extracted primary stem cells from the Achilles tendon of mice for biocompatibility evaluation of the material, and the specific protocol was consistent with a previous report [80]. To infect tumor cells, THP1 cells, and CAFs with lentivirus, they were cultured in 6-well plates until reaching ≤50% confluence. The cells were then transfected with a lenti-B7-H3 vector. Subsequently, the cells were cultured in a medium containing 5 μg/ml Polybrene for 24 h. Stable clones were selected by treating the cells with puromycin for 3 d. For transient transfection, cells were transfected with miRNA mimics using Lipofectamine 3000 reagent (catalog L3000015, Invitrogen, CA).

### In vitro assays for cellular functions

To evaluate cell proliferation, suspended cancer cells were seeded into a 96-well plate at a density of 5 × 10^3^ cells/ml (100 μl/well) and incubated at 37 °C. Subsequently, 10 μl of CCK-8 reagent (catalog KGA9305, KeyGEN, Nanjing, China) was added to each well, followed by a 2-h incubation period. The optical density at 450 nm was then measured using a microplate reader. For assessing cell migration and invasion, Transwell chambers were utilized, with or without Matrigel (Corning) coating as needed. Cancer cells (5 × 10^4^) in 200 μl of serum-free medium were placed in the upper chamber, while 600 μl of medium containing 10% FBS was added to the lower chamber.

### In vitro cytotoxicity assay

To perform in vitro cytotoxicity assay, healthy control peripheral blood mononuclear cells (PBMCs) were obtained and CD8^+^ T cells were isolated using the Dynabeads Human CD8 Selection Kit (catalog 11333D, Invitrogen). The isolated CD8^+^ T cells were cultured in ImmunoCult-XF T cell expansion medium (catalog 10981, STEMCELL Technologies). To activate the T cells, ImmunoCult Human CD3/CD28 T cell activator (catalog 10971, STEMCELL Technologies) was employed. Subsequently, the activated T cells were transferred into a 24-well plate and cocultured with tumor cells at an effector-to-target ratio of 5:1 at 37 °C for 48 h. Flow cytometry analysis was then performed on the T cells to detect apoptosis.

### Quantitative real-time PCR and Western blotting analysis

Total RNA of cells was extracted utilizing TRIzol reagent (catalog KGF5101, KeyGEN, Nanjing, Nanjing). The primers for miR-29a-3p, U6, B7-H3, and glyceraldehyde-3-phosphate dehydrogenase (GAPDH) mRNA reverse transcription were synthesized in KeyGEN (Nanjing, China). Quantitative real-time polymerase chain reaction (qRT-PCR) was performed utilizing the One-Step TB Green PrimeScript RT-PCR Kit II (SYBR Green) (catalog RR086B, TaKaRa, Kyoto, Japan). Primers utilized for gene amplification were in Table S2.

Cellular total proteins were extracted using a lysis buffer, followed by performing SDS-PAGE (polyacrylamide gel electrophoresis) and Western blotting analysis according to established protocols. The primary antibodies used were as follows: B7-H3 (1:1,000 dilution, catalog ab219648, Abcam), COL1A1 (1:1,000 dilution, catalog A24112, Abclonal, Wuhan, China), YAP (1:1,000 dilution, catalog 13584-1-AP, ProteinTech), p-YAP (1:1,000 dilution, catalog 13008, Cell Signaling Technology), and GAPDH (1:2,000 dilution, catalog 60004-1-Ig, ProteinTech). B7-H3 protein levels were standardized to GAPDH.

### In vitro macrophage polarization assay

To induce M2 polarization of THP1 macrophage, 20 ng/ml IL-4 (catalog KGD1203, KeyGENE) was added into the medium for 24 h. In addition, CD86 and CD163 expression was checked by flow cytometry analyses. The antibodies utilized for flow cytometry were as follows: phycoerythrin (PE) anti-CD86 (catalog PE-65165, ProteinTech) and allophycocyanin (APC) anti-CD163 (catalog APC-65169, ProteinTech).

### Immunofluorescence and actin cytoskeleton staining

The expression levels and subcellular location of YAP1 in CAFs were assessed using immunofluorescence assay according to standardized protocols [81]. The primary antibodies utilized were as follows: YAP1 (1:200 dilution, catalog 13584-1-AP, ProteinTech). In addition, CAFs were also subjected to actin cytoskeleton staining using the TRITC phalloidin (catalog 40734ES75, Yeasen). The detailed protocol was described previously [81]. The stained cells were visualized using a fluorescence microscope.

### Preparation of lipo@miR-29a-3p and characterization

According to our previous study [77], the liposomal components [C12-200, cholesterol, 1,2-distearoyl-sn-glycero-3-phosphorylcholine (DSPC), and methoxypolyethylene glycol-dimyristoyl glycerol (mPEG-DMG)] were dissolved in ethanol to achieve a molar ratio of 50:38.5:10:1.5. This mixture was then combined with miR-29a-3p mimics, which had been previously dissolved in a 20 mM citrate buffer at pH 3, through vigorous vortexing. Unencapsulated miR-29a-3p mimics were removed using ultrafiltration centrifugation. The encapsulation efficiency of the liposomes was determined using the Quant-iT RiboGreen RNA assay kit (Molecular Probes, UK) with a spectrofluorometer, setting the excitation wavelength at 480 nm and emission wavelength at 520 nm. Prior to use, the prepared liposomes were diluted in PBS. The entrapment efficiency of the liposomes was also evaluated using the RiboGreen assay.

The hydrodynamic diameter, zeta potential, polydispersity index, and stability of the liposomes were measured using dynamic light scattering (DLS) with a Malvern Zetasizer Nano-ZS, UK. The morphology of the liposomes was examined using TEM. To assess biocompatibility, primary stem cells from the Achilles tendon of mice were cocultured with the liposomes for 1 and 3 d, and cell viability was evaluated using the live/dead staining. Animal biocompatibility is reflected by H&E and Masson staining for tissue sections of heart, liver, spleen, lungs, and kidneys.

Other tests were employed to further test biocompatibility of the materials. Each mouse received liposome injections every 3 d for half a month (a total of 5 injections). Mice were divided into 3 groups: empty liposome group (lipo@miR-ctrl), miR-29a-3p-loaded liposome group (lipo@miR-29a-3p), and normal control group (untreated). After injection, blood samples were collected from all mice within 24 h after surgery and sent to the biochemical analysis laboratory for testing. Peripheral blood samples from mice were collected into anticoagulation tubes and centrifuged at 6,000 rpm for 10 min to isolate the serum. Liver and kidney toxicity were assessed by measuring the serum levels of alanine aminotransferase (ALT), aspartate aminotransferase (AST), creatinine (CREA), and blood urea nitrogen (BUN). These parameters were analyzed using a clinical chemistry analyzer (AU5800, Beckman Coulter).

### In vivo tracking of lipo@miR-29a-3p

To monitor the in vivo distribution of lipo@miR-29a-3p, liposomes were tagged with DIR (catalog D12731, Invitrogen). For this process, DIR-labeled liposomes at a dose of 0.1 mg/kg were administered intravenously to mice bearing tumors. At 24 h after injection, the mice were euthanized, and their organs were immediately harvested for analysis using an in vivo imaging system (Invivo Smart-LF, Vieworks, South Korea). This methodology allowed for the precise evaluation of the biodistribution of the liposomal formulation within the murine model.

### Animal models and analysis of mouse tumor tissues

Female BALB/C mice (5 to 6 weeks old) were obtained from Shanghai Laboratory Animal Center (SLAC). To establish the mouse triple-negative breast cancer (TNBC) models, 4T1 cells (5 × 10^6^) were subcutaneously injected into the flanks of BALB/C mice. Tumors were monitored and regularly measured with calipers every 2 to 3 d. When tumors reached about 100 mm^3^ in volume, mice bearing tumors were randomized into 2 groups (*n* = 5), including lipo@crtl and lipo@miR-29a-3p groups. The dose used for the treatment of lipo@miR-29a-3P was 100 μl of liposome at the concentration of 0.1 mg/kg. The treatment regimen was continued for an additional 21 d or until the tumors reached a length of 20 mm. The mice were humanely euthanized using carbon dioxide in the Euthanex Chamber. Subsequently, the tumors were excised from the anesthetized animals, documented, and weighed. Detailed breeding conditions could be found in the previous study [82]. Removed tumors were submitted for scRNA-seq, mass cytometry, immunofluorescence, and flow cytometry analyses. Antibodies used for immunofluorescence were as follows: CD86 (1:200 dilution, catalog 13395-1-AP, ProteinTech), CD163 (1:200 dilution, catalog gb15340, Servicebio), F4/80 (1:200 dilution, catalog gb113373, Servicebio), Ki-67 (1:200 dilution, catalog gb111499, Servicebio), CD8 (1:200 dilution, catalog gb15068, Servicebio), and B7-H3 (1:200 dilution, catalog sc-376535, Santa Cruz Biotechnology). We counted the number of target cells in the unit area or fluorescence intensity for statistics (*n* = 5). The description for flow cytometry was stated as previously [82], and antibodies used for flow cytometry were as follows: fluorescein isothiocyanate (FITC) anti-F4/80 (catalog E-AB-F0995C, Elabscience), APC anti-CD163 (catalog E-AB-F1295E, Elabscience), PE anti-CD86 (catalog E-AB-F0994D, Elabscience), PE anti-CD8 (catalog E-AB-F1104D, Elabscience), and FITC anti-PD-1 (catalog E-AB-F1131C, Elabscience).

We also investigated the therapeutic effectiveness of the combination of lipo@miR-29a-3p and anti-B7-H3 therapy. Similar to the method described above, when tumors reached about 100 mm^3^ in volume, BALB/C mice bearing 4T1 cells were randomized into 4 groups (*n* = 5), including lipo@crtl, lipo@miR-29a-3p, lipo@crtl + anti-B7-H3, and lipo@miR-29a-3p + anti-B7-H3 groups. The B7-H3 in vivo monoclonal antibody (catalog BE0124, BioXCell) was dissolved in InVivoPure pH 7.0 dilution buffer, and 200 μg was administered intraperitoneally 3 times a week. In addition, paraffin-embedded tumor samples from previous mouse exercise models [83] were submitted for miR-29a-3p immunofluorescence staining.

### scRNA-seq analysis

For scRNA-seq, a small piece of 5 tumor tissues from 2 groups was taken and put into the tissue storage solution (catalog cytoG100, BioMedicine Technology). scRNA-seq was performed by BioMedicine Technology. CellRanger was used to perform barcode processing and generate gene count profiles. The Seurat (4.3.01, http://satijalab.org/seurat/>) R toolkit was used to perform all analyses [84]. Cells were removed if the expression of mitochondrial genes was greater than 10% or with detected genes less than 200 or greater than 5,000. The “RunHarmony” function in the R package harmony [85] was used to minimize the technical batch effects among individuals and experiments. To minimize dimensionality, principal components analysis (PCA) [86] was performed based on the top 4,000 variable genes. The dimensionality of the scaled integrated data matrix was then further reduced to 2-dimensional space and visualized using t-distributed stochastic neighbor embedding (t-SNE) [87], based on the top 30 PCs. A shared nearest neighbor [88] modularity optimization-based clustering approach with a resolution was used to identify the cell clusters. Then, 15,526 cells were unsupervised clustered into 22 clusters with a resolution of 1.

To display the expression distribution of B7-H3, the scRNA-seq dataset of 10 BRCAs was obtained from our previous study [70], and processed and analyzed using the same methods and parameter values as the mouse single-cell dataset. Then, 36,613 cells were unsupervised classified into 24 clusters with a resolution of 1.

The proliferation score of each tumor cell was calculated by using a signature of 10 highly expressed genes in cycling cells (ASPM, CENPE, CENPF, DLGAP5, MKI67, NUSAP1, PCLAF, STMN1, TOP2A, and TUBB) based on the method of van Galen et al. [89]. First, we selected the 100 genes with the most similar average expression levels as a background gene set for each of these genes. The background gene set average expression was subtracted from each signature gene, and the average of these values for all signature genes was determined as the proliferation score. In addition, the aggressiveness score of tumor cells were assessed by the “AddModuleScore” function in the Seurat package based on the migration and invasion signatures obtained from the study of Valastyan and Weinberg [90].

In addition, by utilizing the “AddModuleScore” function in the Seurat package, we assessed the M1 and M2 scores of macrophages and activated fibroblasts based on the conventional signatures: M2: FN1, CD163, CD206, CD209, FIZZ1, CCL1, CCL17, CCL18, CCL22, CCL24, CXCL13, VEGFA, VEGFB, VEGFC; M1: IL23, TNF, CXCL9, CXCL10, CXCL11, CD86, IL1A, IL1B, IL6, CCL5, IRF5, IRF1, CD40, IDO1, KYNU, and CCR7; activated fibroblasts: CD276, PDGFRA, PDGFRB, FAP, NOTCH3, HES4, THY1, TWIST2, CREB3L1, COL1A1, COL1A2, COL3A1, COL4A2, MMP11, and MMP14.

### Cell–cell communication analysis

According to the application of the CellPhoneDB software [91,92] in investigating the potential functional mechanisms of crucial cell subpopulations [93], we constructed the molecular interaction networks to describe the holistic view of the TME changes induced by miR-29a-3p. The ligand–receptor pairs with a *P* value of <0.05 were retained for evaluating the relationships among different cell clusters.

### Mass cytometry

Mass cytometry, also known as CyTOF, was conducted utilizing protocols adapted from previous methodologies [94]. In brief, cell suspension from tumor samples underwent a 15-min exposure to stable palladium isotopes using Maxpar Barcode Perm Buffer (catalog 201067, Fluidigm, USA) to achieve labeling with distinct combinations of isotopically pure palladium ions complexed with isothiocyanobenzyl-EDTA. Subsequent to labeling, cells were treated with Fc Receptor Blocking Solution (catalog abs9477, Absin, China) to prevent nonspecific binding, followed by a 30-min incubation with a cocktail of antibodies. Following a series of washes, cells were permeabilized using methanol at 4 °C for 10 min and subsequently stained with intracellular markers for 1 h. The stained cells were then incubated overnight with a 191Ir/193Ir iridium intercalator solution (DVS Sciences, Toronto, Canada) in 4% paraformaldehyde. Calibration beads were introduced prior to analysis on a Helios mass cytometer (Helios Mass Cytometer, Fluidigm, USA). A target of approximately 0.5 × 10^6^ cells was acquired from each sample at a flow rate of 400 to 500 events per second. Data analysis was conducted using Cytobank v8.1 (Beckman Coulter), employing the vi-SNE algorithm for dimensionality reduction (default parameters) and visualization. A panel comprising 24 antibodies was used throughout the experimental process (see Table S3 for details).

### Statistical analysis

Statistical analysis and data visualization were conducted using R language version 4.0.2 and GraphPad Prism version 6.0. Continuous variables between 2 groups were compared using Student’s *t* test or Mann–Whitney test as appropriate. Differences among multiple groups were assessed using one-way analysis of variance (ANOVA) or Kruskal–Wallis test with multiple comparisons as applicable. Categorical variables were analyzed using the chi-square test or Fisher exact probability test based on the conditions. Correlation between 2 variables was evaluated using Pearson or Spearman correlation test depending on the conditions. The prognostic significance of categorical variables was determined using the log-rank test. A *P* value of <0.05 was considered statistically significant and denoted as *P < 0.05; **P < 0.01; ***P < 0.001 for clarity.

## Ethical Approval

Ethical approval for the study of TMAs was granted by the Outdo Biotech Clinical Research Ethics Committee. Ethical approval for the in-house cohort’s recruitment was granted by Clinical Research Ethics Committees at Wuxi Maternal and Child Health Care Hospital. Animal experiments were granted by Ethics Committees at Nanjing Medical University.

## Data Availability

All data supporting the results of this study are shown in this published article and supplementary documents. In addition, original omics data for bioinformatics analysis could be obtained from corresponding platforms.
