# Supplementary material for: Altered Atlas of Exercise-Responsive MicroRNAs Revealing miR-29a-3p Attacks Armored and Cold Tumors and Boosts Anti-B7-H3 Therapy
Source: Research (Wash D C). 2025 Jan 22;8:0590. doi: 10.34133/research.0590 (PMC11751204; doi:10.34133/research.0590)
Supplement: Supplementary 1 — Tables S1 to S3 Figs. S1 to S14 [file research.0590.f1.docx]

**Supplementary Table**

**Supplementary Table 1. Summary of included public and in-house cohorts.**

| Dataset | Cancer type | Case | Source |
| --- | --- | --- | --- |
| TCGA | solid cancer types | 9363 | <https://xenabrowser.net/datapages/> |
| scRNA-seq dataset | breast cancer | 10 | doi: 10.1038/s41416-023-02432-6 |
| Exercise cohort | breast cancer | 24 | [in-house](https://www.ncbi.nlm.nih.gov/gds) |
| HBreD140Su03 | breast cancer | 120 | [in-house](https://www.ncbi.nlm.nih.gov/gds) |
| HBreD077Su01 | normal breast tissue | 70 | [in-house](https://www.ncbi.nlm.nih.gov/gds) |
| HLugA150CS01 | lung adenocarcinoma | 68 | [in-house](https://www.ncbi.nlm.nih.gov/gds) |
| HMelC112CD01 | melanoma | 92 | [in-house](https://www.ncbi.nlm.nih.gov/gds) |

**Supplementary Table 2. The sequence of miRNA, primers, and FISH probes.**

| Name | Sequence (5'-3') |
| --- | --- |
| hsa-miR-29a-3p mimic | UAGCACCAUCUGAAAUCGGUUA |
| hsa-miR-ctrl | CAGUACUUUUGUGUAGUACAAA |
| B7-H3 | F-AGCACTGTGGTTCTGCCTCACA  R- CACCAGCTGTTTGGTATCTGTCAG |
| GAPDH | F- AGATCATCAGCAATGCCTCCT  R- TGAGTCCTTCCACGATACCAA |
| hsa-miR-29a-3p | F-CTCAACTGGTGTCGTGGAGTCGGCAATTCAGTTGAGTAACCGAT  R-ACACTCCAGCTGGGTAGCACCATCTGAAAT |
| hsa-U6 | F-CTCGCTTCGGCAGCACA  R-AACGCTTCACGAATTTGCGT |
| mmu-miR-29a-3p | UAGCACCAUCUGAAAUCGGUUA |
| probe-miR-29a-3p | TAACCGATTTCAGATGGTGCTA |
| double-labeled probes | DIG-ttATGATGATGTATGATGATGT |

**Supplementary Table 3. A list of the antibodies and reagents used mass cytometry.**

| Catalog | Product | Company |
| --- | --- | --- |
| 3145002C | CD4 | Fluidigm |
| 3089005C | CD45 | Fluidigm |
| 3152004C | CD3e | Fluidigm |
| 3172016C | CD86 | Fluidigm |
| 3146008C | F4/80 | Fluidigm |
| 3209003C | CD11b | Fluidigm |
| 3141005C | Ly-6G/C | Fluidigm |
| 3169021C | CD206 | Fluidigm |
| 3159006C | CD279 (PD-1) | Fluidigm |
| 3170002C | CD161 (NK1.1) | Fluidigm |
| 3160011C | FITC | Fluidigm |
| 3176007C | APC | Fluidigm |
| 201064 | Cisplatin | Fluidigm |
| 201192A | Intercalator-Ir | Fluidigm |
| 201068 | Maxpar Cell Staining Buffer (500 ML) | Fluidigm |
| 201067 | Maxpar Fix and Perm Buffer (100 ML) | Fluidigm |
| 201069 | Maxpar Water (500 ML) | Fluidigm |
| 201244 | Maxpar Cell Acquisition Solution Plus for CyTOF XT-1L | Fluidigm |
| 201063 | Maxpar Nuclear Antigen Staining Buffer Set | Fluidigm |

**Supplementary Figure**

**
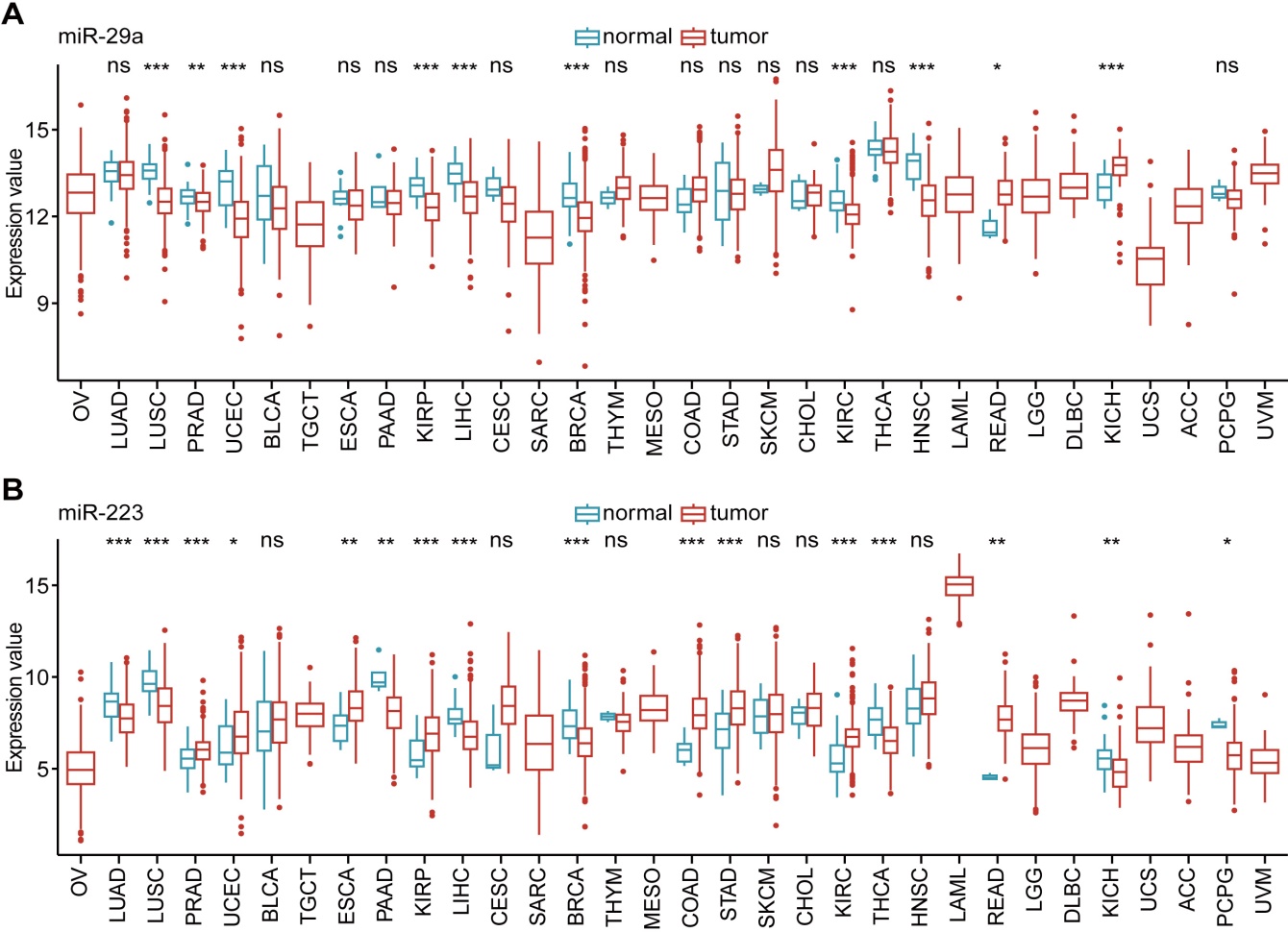
**

**Supplementary Figure 1. Expression levels of candidate exercise-responsive miRNAs in pan-cancer.** (A) Expression levels of miR-29a in pan-cancer in the TCGA dataset. (B) Expression levels of miR-223 in pan-cancer in the TCGA dataset. ns, no significance, *P < 0.05, **P < 0.01, ***P < 0.001.


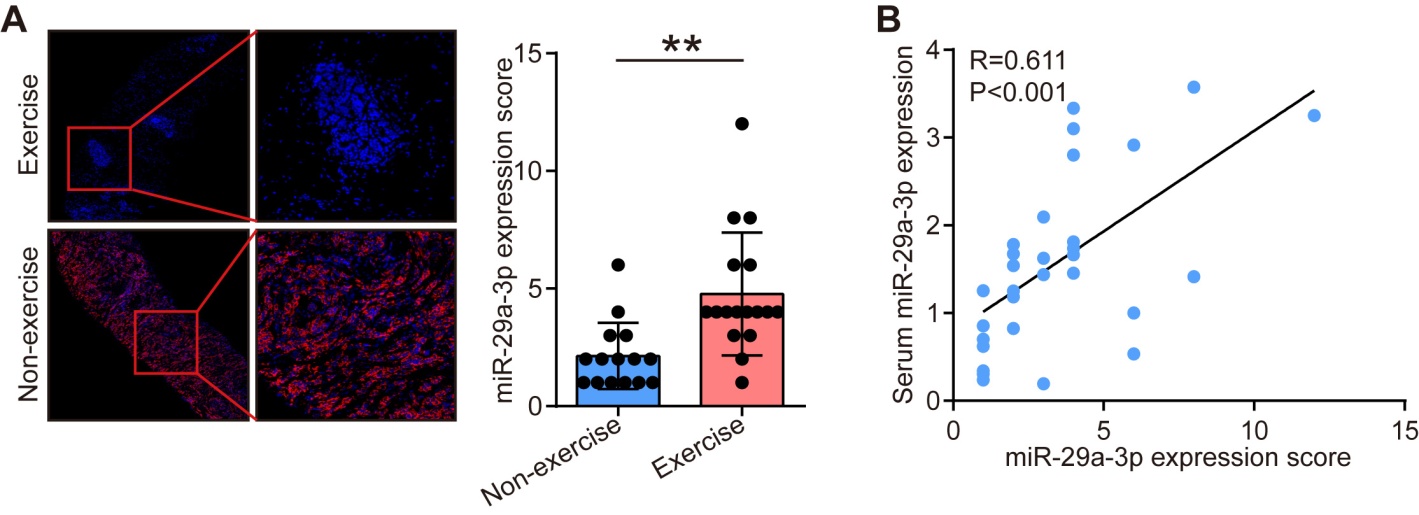


**Supplementary Figure 2. Expression of miR-29a-3p in tumor tissues from patients with or without exercise habit.** (A) Representative images showing miR-29a-3p expression in tumor and para-tumor tissues, along with semi-quantitative analysis. Total original magnification, 50× (left) and 200× (right). Data are presented as mean ± SD. Significance was calculated with Mann-Whitney test. ns, no significance, *P < 0.05. (B) Correlation of tumoral miR-29a-3p and serum miR-29a-3p expression. Significance was calculated with Spearman test.


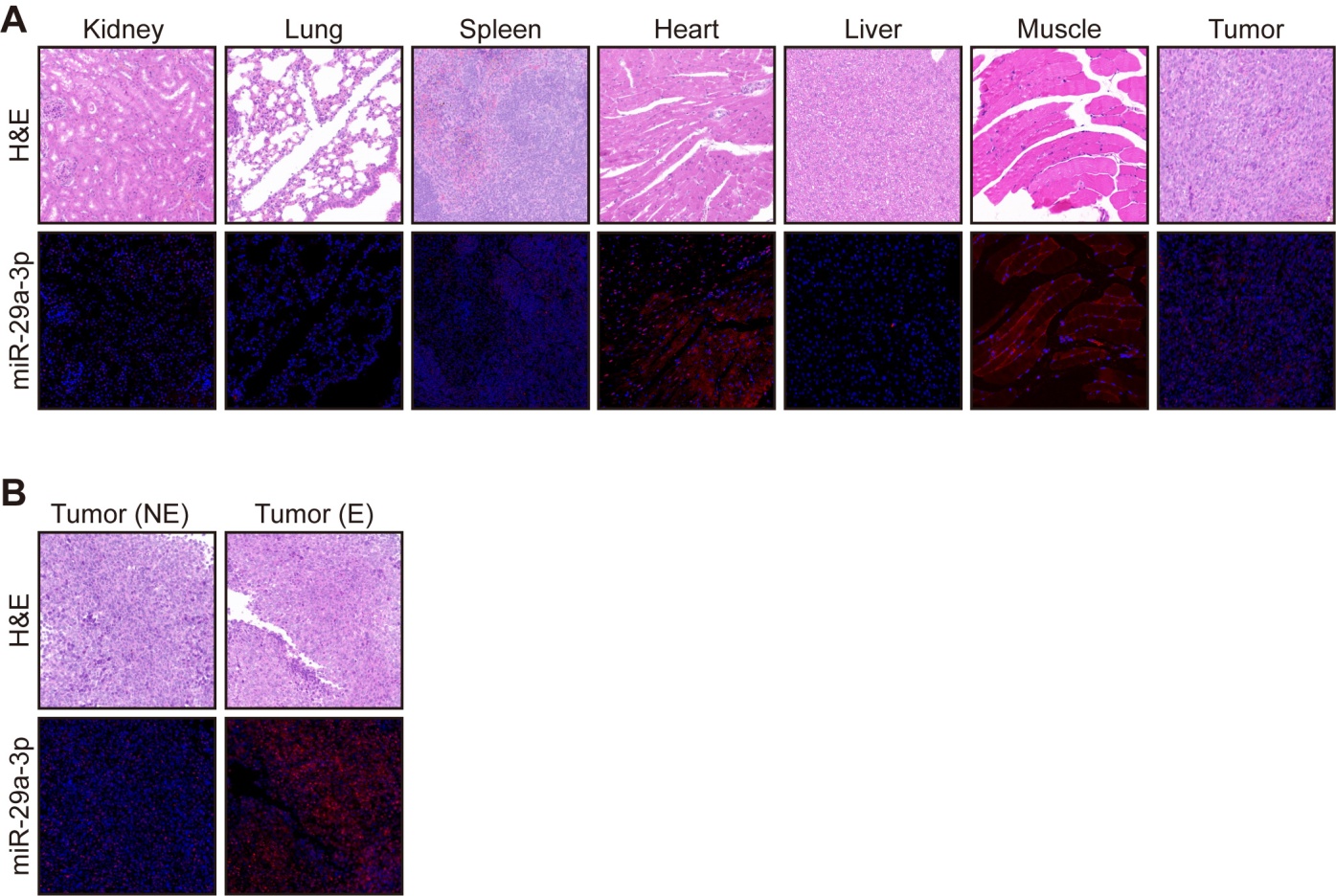


**Supplementary Figure 3. Expression of miR-29a-3p in various organs.** (A) Expression of miR-29a-3p in mouse kidney, lung, spleen, heart, liver, muscle, and tumor tissues. (B) Expression of miR-29a-3p in tumor tissues from mouse with or without exercise. NE, non-exercise; E, exercise.


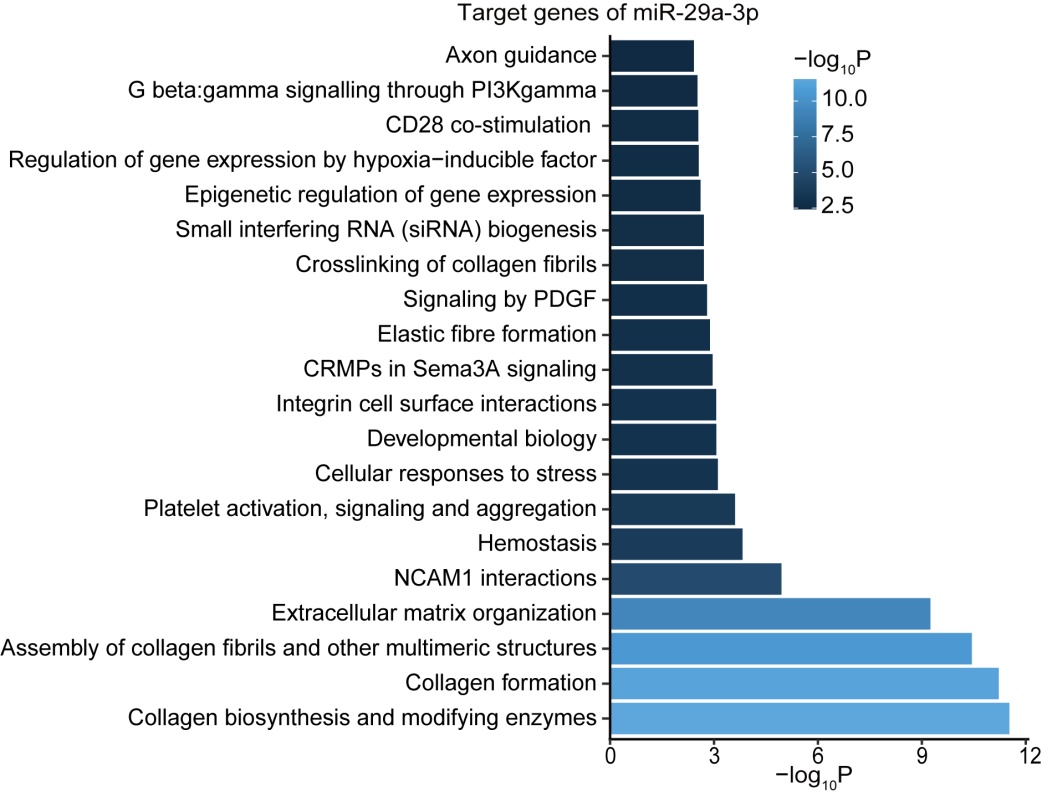


**Supplementary Figure 4. Enrichment of target genes intersected by miRDB and TargetScan using gene sets derived from the Reactome pathway database.**

**
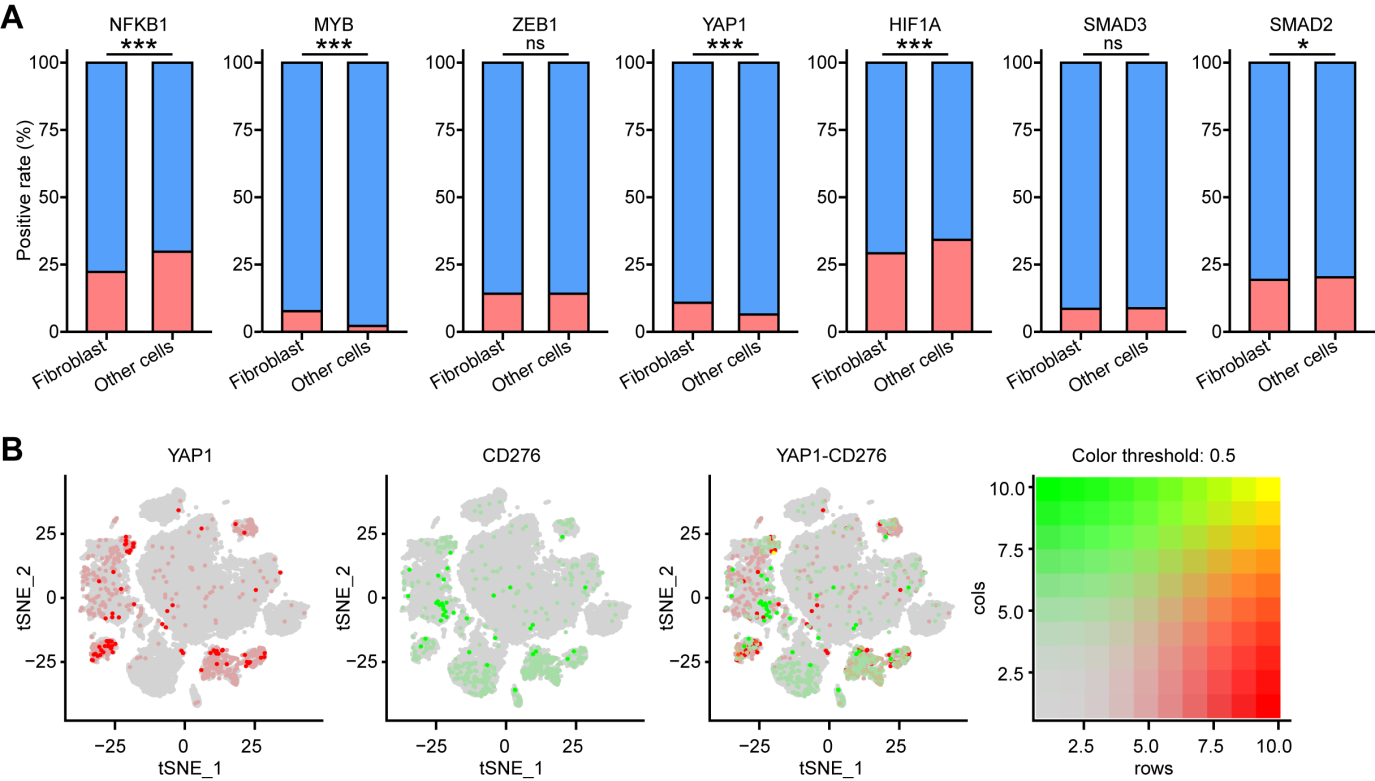
**

**Supplementary Figure 5. Correaltions between B7-H3 and transcriptional factors for COL1A1.** (A) Expression of transcriptional factors for COL1A1 in fibroblasts and other cells. Significance was calculated with Chi-square test. ns, no significance, *P < 0.05, ***P < 0.001. (B) Expression of B7-H3 (gray to red) and YAP1 (gray to green) on overlaid on the t-SNE representation.


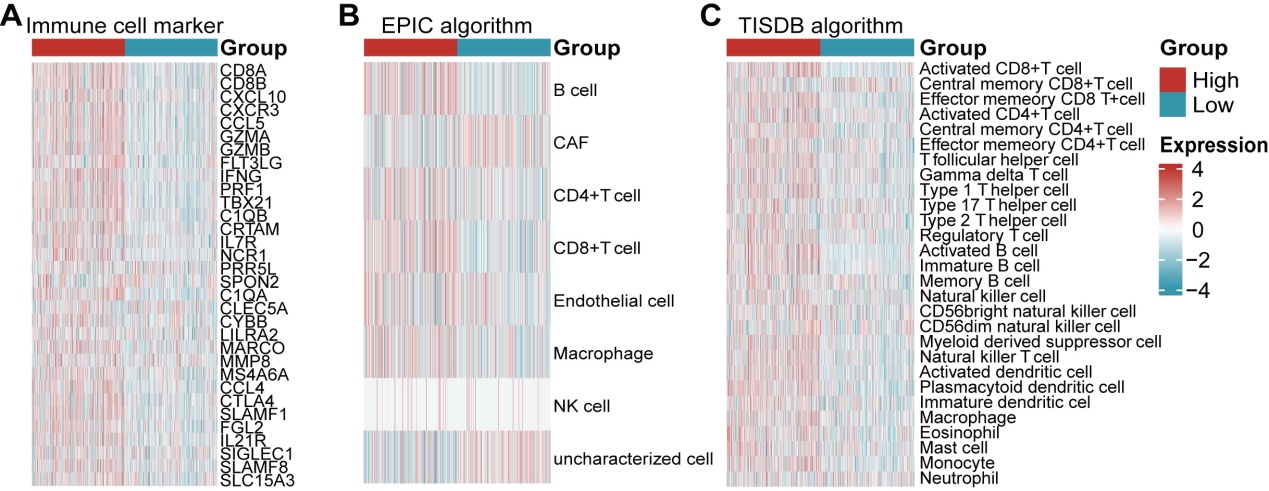


**Supplementary Figure 6. miR-29a-3p was associated with the inflamed TME (supplement).** (A) Expression levels of immune cells in the high- and low- miR-29a-3p groups in BRCA. (B, C) Levels of immune cells in the high- and low- miR-29a-3p groups in BRCA estimated by the EPIC and the TISDB algorithms.

**
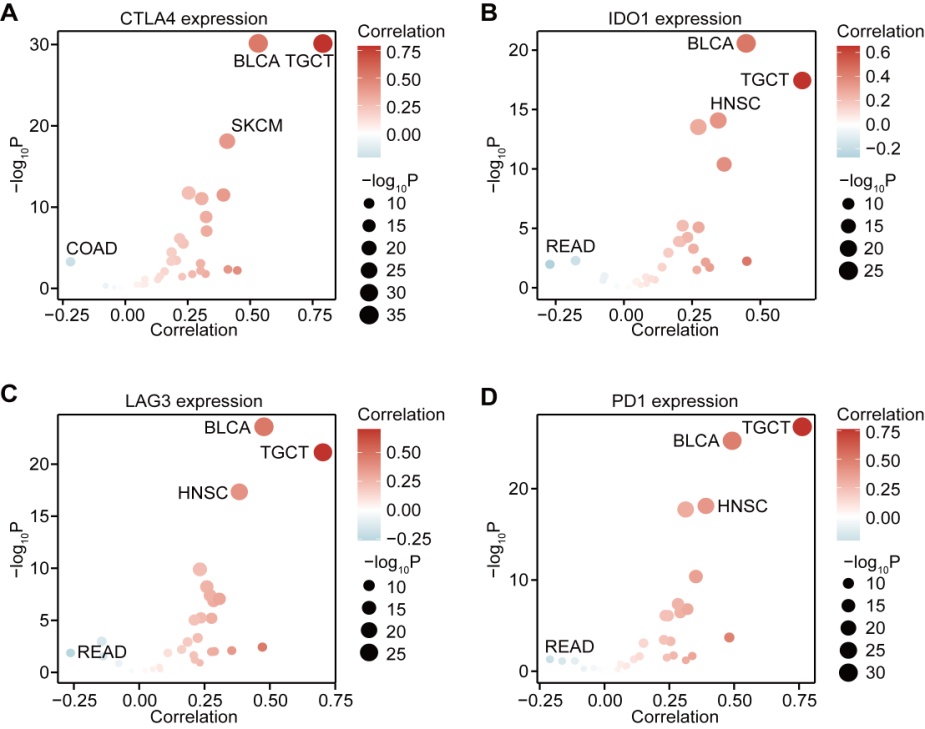
**

**Supplementary Figure 7. The correlation between miR-29a-3p and immune checkpoints expression in pan-cancer.** (A) CTLA4, (B) IDO1, (C) LAG3, and (D) PD1. Data was obtained from the TCGA database. Significance was calculated with Pearson test.

**
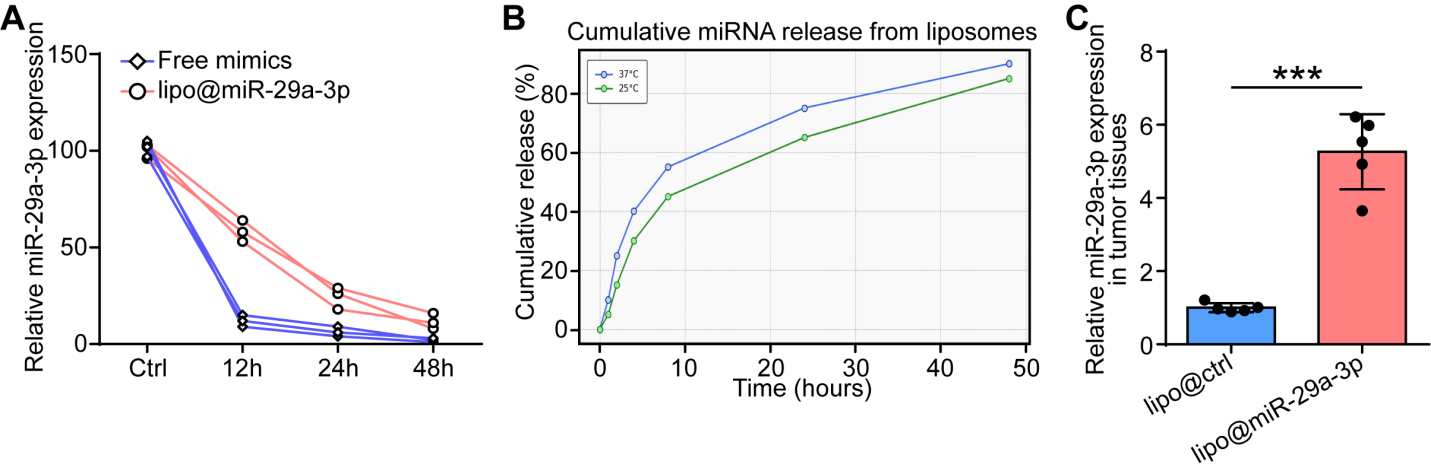
**

**Supplementary Figure 8. The characterizations of the lipo@miR-29a-3p *in vitro* and *in vivo*.** In vitro release experiments showing that (A) lipo@miR-29a-3p exhibited slow and stable miRNA release under specific conditions and (B) miRNA release from this liposomal system was stable at different temperatures. (C) Expression of miR-29a-3p in tuomr tissues from mouse receiving lipo@ctrl or lipo@miR-29a-3p therapies. Significance was calculated with Student t test. ***P < 0.001.

**
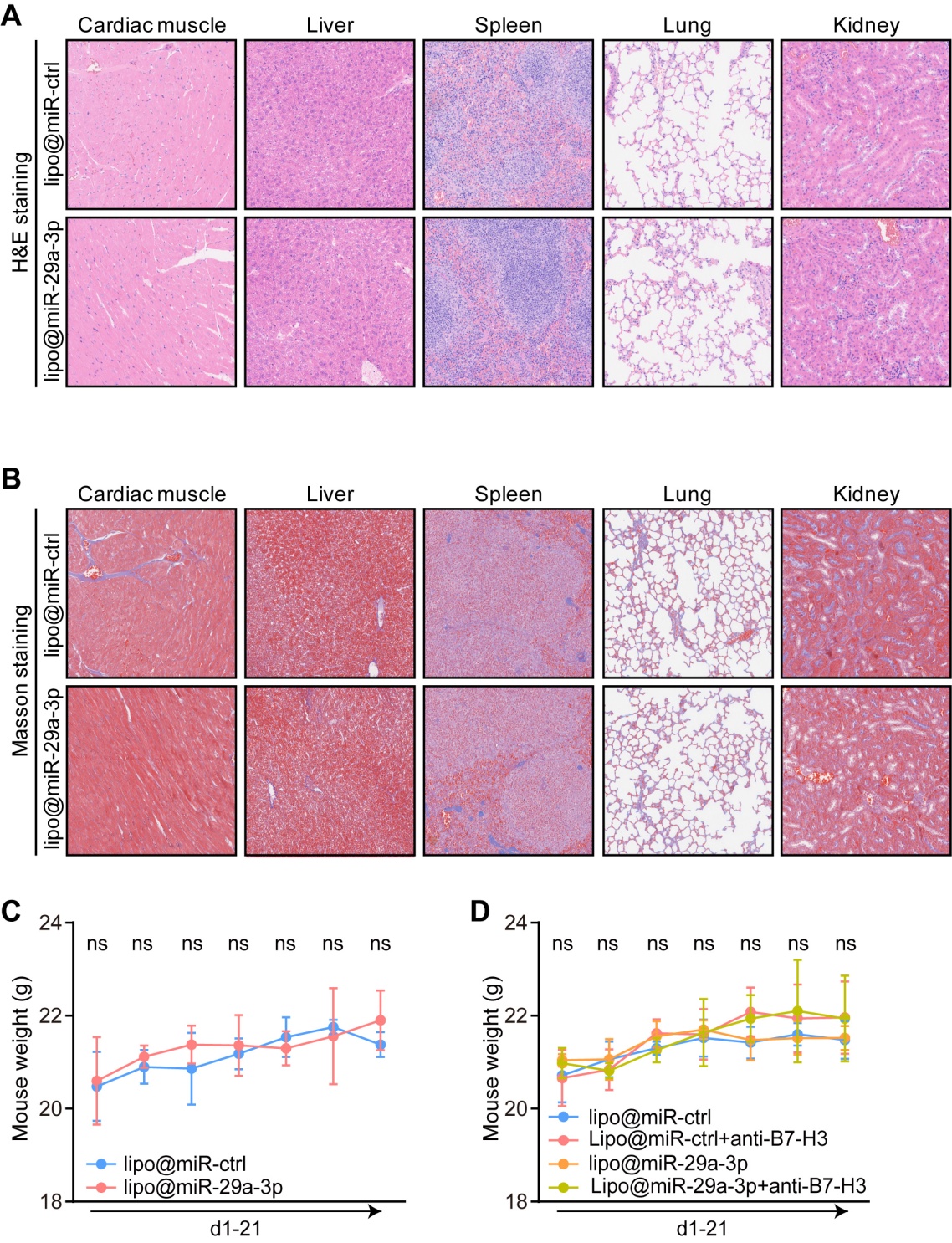
**

**Supplementary Figure 9. Biocompatible and tolerance of lipo@miR-29a-3p *in vivo*.** (A) HE and (B) Masson staining of heart, liver, spleen, lung and kidney tissues from mice receiving lipo@miR-ctrl and lipo@miR-29a-3p therapy. (C) Effect of lipo@miR-29a-3p on mouse weight in balb/c mice bearing 4T1 cells. Data presented as mean ± SD. Significance was calculated with Student’s t test. ns: no significance. (D) Effect of B7-H3 mAb, lipo@miR-29a-3p, and combination on mouse weight in balb/c mice bearing 4T1 cells and quantitative analysis. Data presented as mean ± SD. Significance was calculated with 1-way ANOVA with Tukey’s multiple-comparison test. ns, no significance.

**
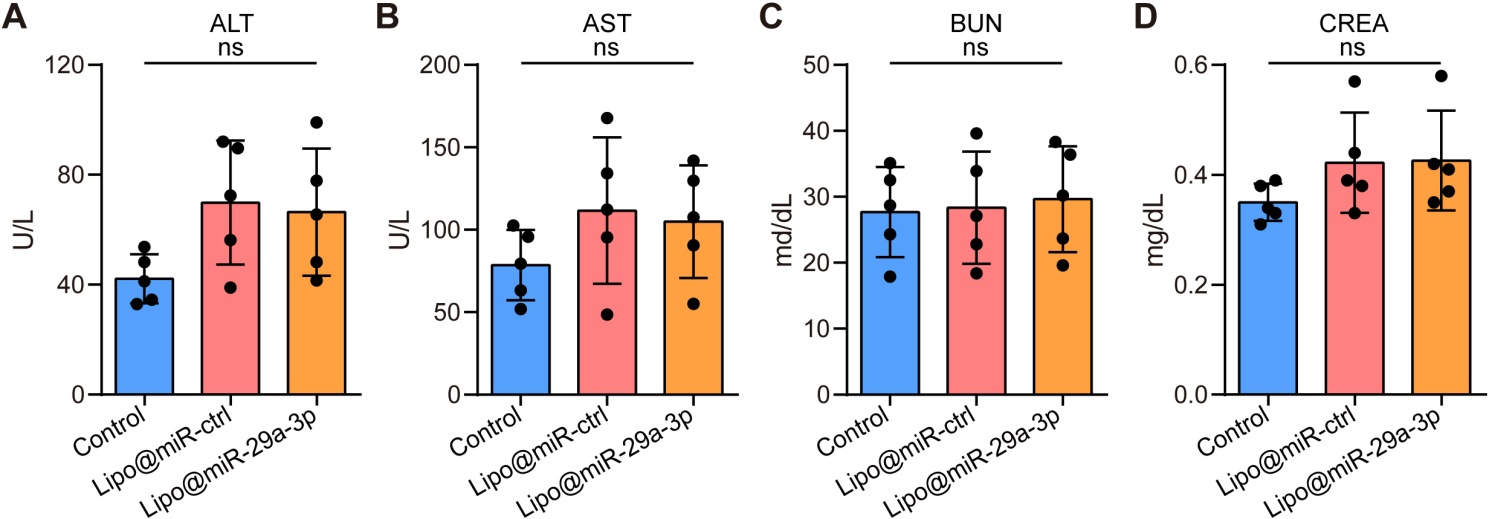
**

**Supplementary Figure 10. Blood biochemical analysis of liver and kidney function in mice treated with liposomal formulations.** Serum levels of alanine aminotransferase (ALT), aspartate aminotransferase (AST), blood urea nitrogen (BUN), and creatinine (CREA) were measured in balb/c mice bearing 4T1 cells. Data presented as mean ± SD. Significance was calculated with 1-way ANOVA with Tukey’s multiple-comparison test. ns, no significance.

**
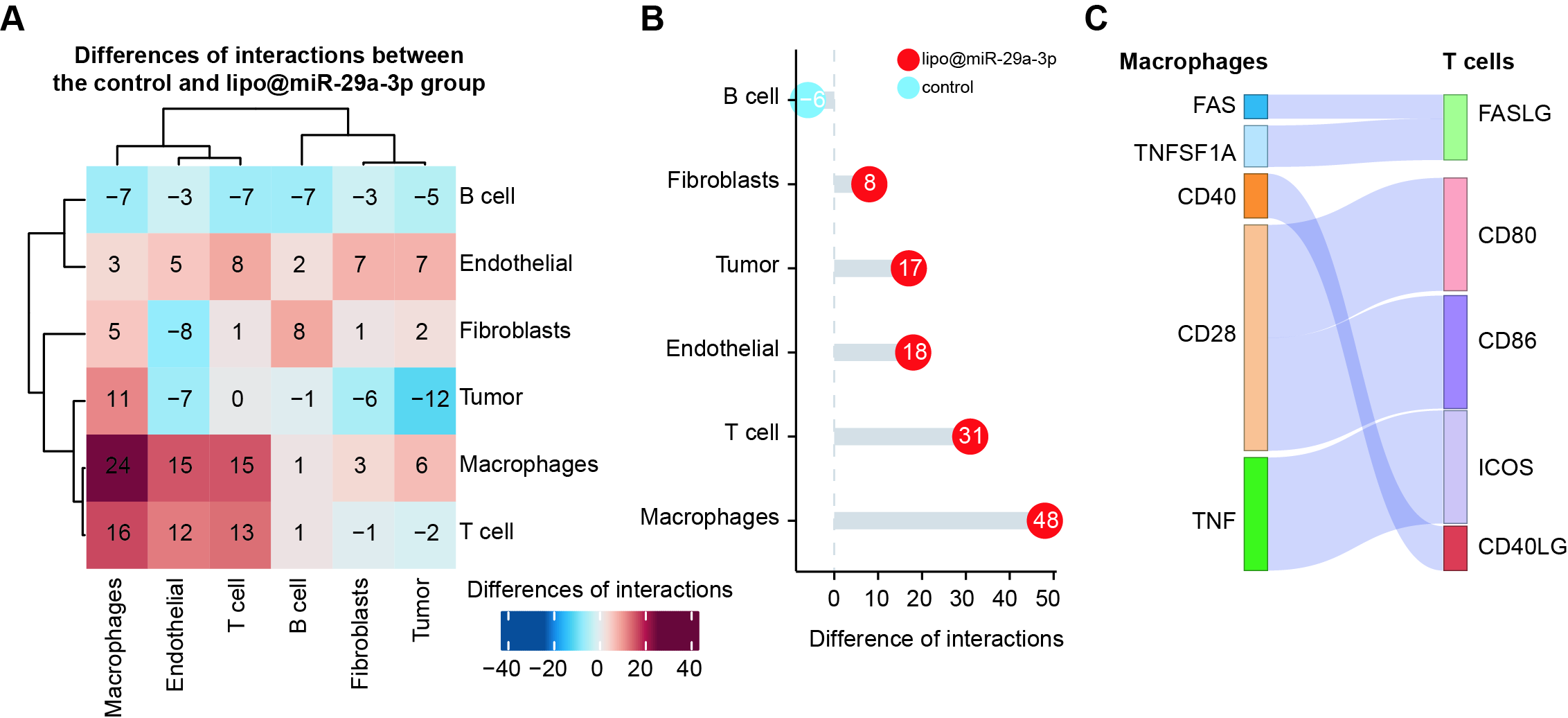
**

**Supplementary Figure 11. Cell-cell communications among cell types in the lipo@miR-ctrl and lipo@miR-29a-3p group, respectively.** (A) Heatmap showing the differences of interactions between the lipo@miR-ctrl and lipo@miR-29a-3p group. Red represents the interactions between the two cell types were more enriched in the lipo@miR-29a-3p group than in the lipo@miR-ctrl. Blue represents the interactions between the two cell types were more enriched in the lipo@miR-ctrl group than in the lipo@miR-29a-3p. (B) The difference of the number of ligand-receptor interactions between macrophages and other cell types between the lipo@miR-ctrl and lipo@miR-29a-3p group. (C) The interactions between macrophages and T cells in the lipo@miR-29a-3p group.

**
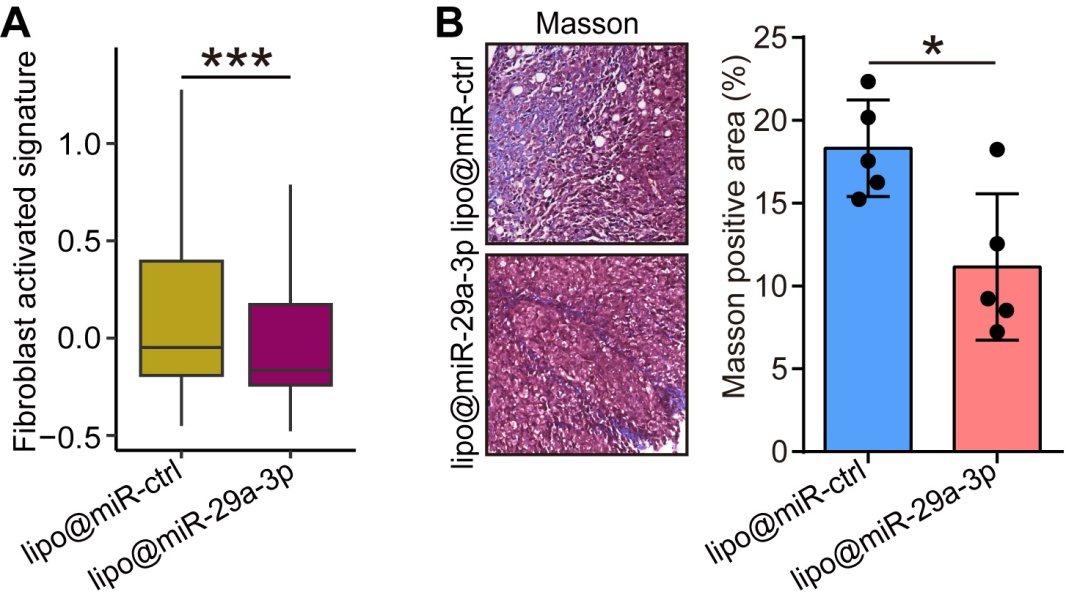
**

**Supplementary Figure 12. Lipo@miR-29a-3p decreased collagen deposition. (A)** Level of activated signature in fibroblast from tumor tissues with lipo@miR-ctrl and lipo@miR-29a-3p treatment. Significance was calculated with Student’s t test. ***P < 0.001. (B) Representative images showing collagen area determined by Masson staining in tumor tissues with lipo@miR-ctrl and lipo@miR-29a-3p treatment, along with quantitative analysis. Total original magnification, 200×. Significance was calculated with Student’s t test. *P < 0.05.

**
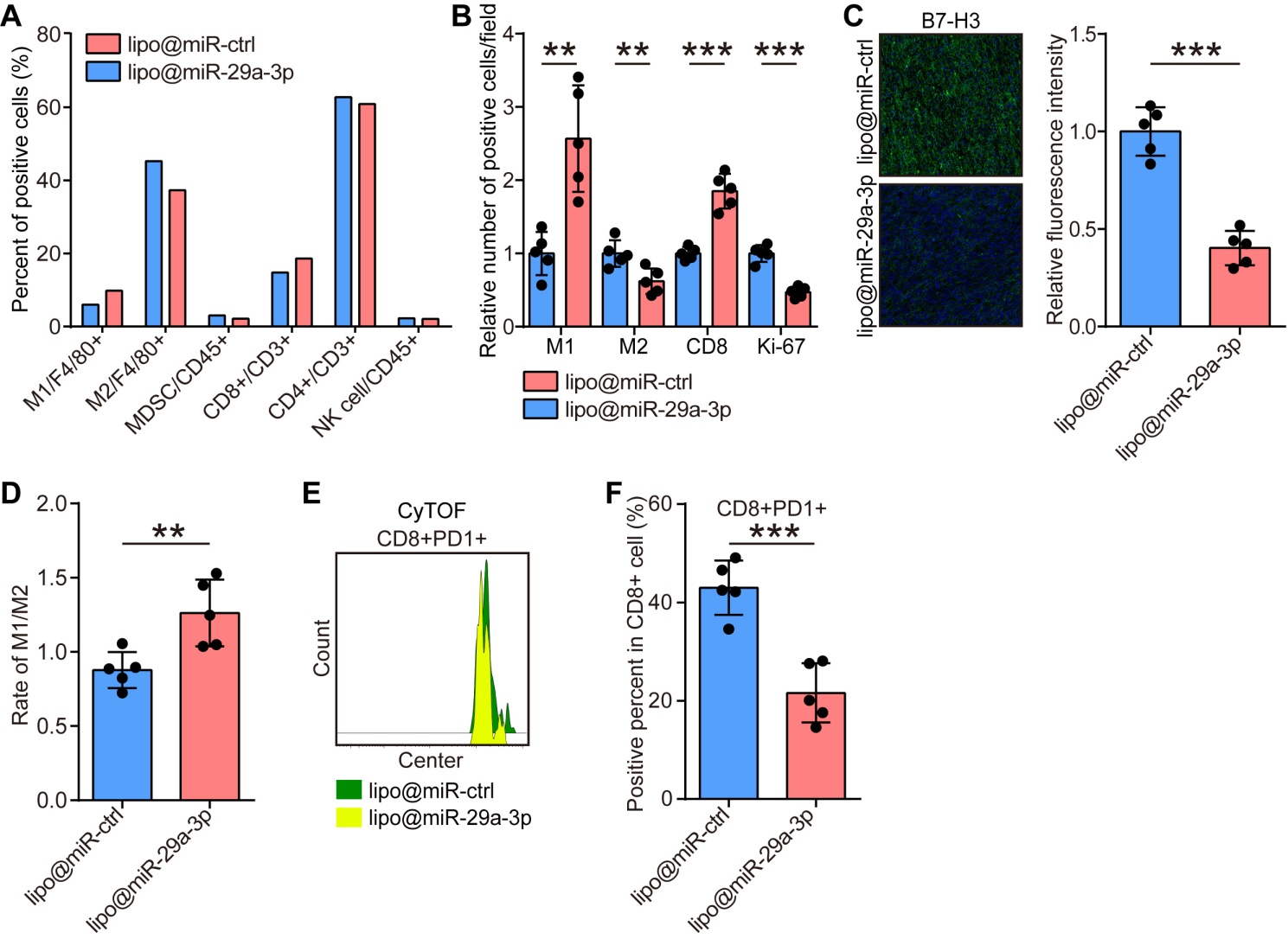
**

**Supplementary Figure 13. Lipo@miR-29a-3p activated TME (supplement).** (A) Percent of various immune cells in tumor tissues with lipo@miR-ctrl and lipo@miR-29a-3p treatment. (B) Quantitative analysis of immunofluorescence assays for Figure 7G. Data presented as mean ± SD. Significance was calculated with Student’s t test. **P < 0.01, ***P < 0.001. (C) The expression of B7-H3 was examined by immunofluorescence assay. Total original magnification, 200×. Significance was calculated with Student’s t test. ***P < 0.001. (D) The rate of M1/M2 macrophages in tumor tissues with lipo@miR-ctrl and lipo@miR-29a-3p treatment was examined by flow cytometry. Data are presented as mean ± SD. Significance was calculated with student t test. **P < 0.01. (E) CyTOF showing decreased exhausted CD8^+^T cells in tumors receiving lipo@miR-29a-3p treatment. (F) The level of exhausted CD8^+^T cells in tumor tissues with lipo@miR-ctrl and lipo@miR-29a-3p treatment was examined by flow cytometry. Data are presented as mean ± SD. Significance was calculated with student t test. **P < 0.01.

**
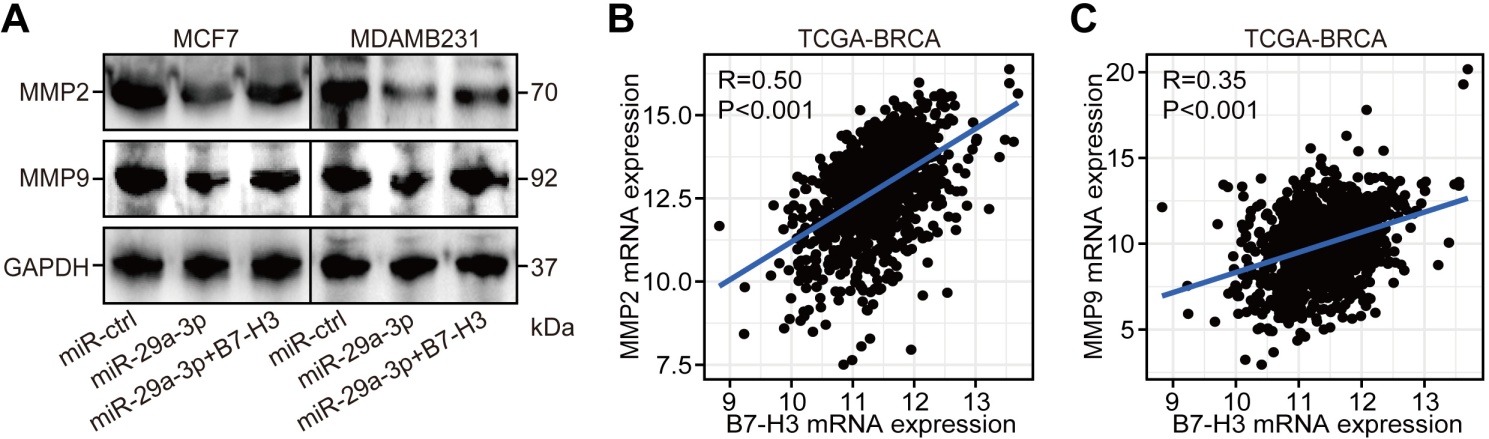
**

**Supplementary Figure 14. Correlation between B7-H3 and MMP2 & MMP9 in BRCA.** (A) The expression of MMP2 and MMP9 in control, miR-29a-3p-overexpressed, and B7-H3-rescured BRCA cells was assessed by Western blotting assay. (B) The correlation between B7-H3 and MMP2 in BRCA. Data was obtained from the TCGA database. Significance was calculated with Pearson test. (C) The correlation between B7-H3 and MMP9 in BRCA. Data was obtained from the TCGA database. Significance was calculated with Pearson test.
